# Shedding light on neural learning to rank models for anticancer drug prioritization

**DOI:** 10.1371/journal.pone.0345854

**Published:** 2026-08-13

**Authors:** Faraz Sarmeili, Benyamin Ghahremani-Nezhad, Mohammad Khalilpour, Karim Abbasi, Rassoul Dinarvand, Hamid R. Rabiee

**Affiliations:** 1 Department of Pharmaceutics, Faculty of Pharmacy, Tehran University of Medical Sciences, Tehran, Iran; 2 Department of Computer Engineering, Sharif University of Technology, Tehran, Iran; 3 Department of Computer Engineering, Amirkabir University of Technology, Tehran, Iran; 4 Mosaheb Institute for Mathematical Research, Kharazmi University, Tehran, Iran; 5 Nanotechnology Research Centre, Faculty of Pharmacy, Tehran University of Medical Sciences, Tehran, Iran; Jamia Millia Islamia Central University: Jamia Millia Islamia, INDIA

## Abstract

Learning to Rank (LeToR) methods have gained increasing attention in drug response prediction, offering a direct way to prioritize effective treatments for cancer cell lines. In this study, we systematically benchmark six ranking loss functions, including state-of-the-art listwise methods, and five types of molecular representations across two large-scale drug screening datasets, CTRP and PRISM. Using high-dimensional gene expression profiles and various drug fingerprints and descriptors, we evaluated models under multiple validation setups and ranking metrics. Our results demonstrate that listwise loss functions such as LambdaLoss and LambdaRank consistently excel in both early and overall ranking quality. Additionally, combining molecular fingerprints with physicochemical descriptors yielded improved performance. A novel attention-based mechanism and a modified version of RankingSHAP were integrated to enhance interpretability, uncovering key genes and substructures aligned with known biological insights. The explainability pipeline successfully distinguished estrogen receptor-positive (ER⁺) and estrogen receptor-negative (ER^−^) breast cancer subtypes. The model successfully identified critical substructures in docetaxel, an FDA-approved therapy, and triptolide, which is currently undergoing clinical evaluation for breast cancer. These findings are consistent with established structure-activity relationship (SAR) data. Overall, this study presents a comprehensive evaluation framework and underscores the importance of carefully selecting loss functions and feature representations when developing robust and interpretable drug-ranking systems.

## 1 Introduction

One of the primary causes of death worldwide is cancer. Although the screening, diagnosis, treatment, and prevention strategies for cancer have significantly improved in recent years, cancer incidence and mortality continue to rise annually, contributing to a growing global health and economic burden. One reason for these increases is the complex and heterogeneous nature of cancer [1–3].

Cancer treatment complexity stems from the evolving genetic diversity of cancer cells, leading to varied drug responses among patients [4,5]. Therefore, precision medicine appears to be a suitable strategy for treating this disease. Consequently, cancer drugs are becoming more targeted, and specific biomarkers increasingly guide therapeutic guidelines [6,7]. A well-known example is the expression of the *HER2* receptor in breast cancer (BC), where the ASCO-CAP guidelines recommend different chemotherapeutic regimens depending on *HER2* status. These biomarker-driven treatment protocols are expected to become more widespread for other types of cancer [8].

On the other hand, the process of drug development is costly and lengthy, according to a recent article by Sertakya et al. [9]. The cost of developing a new drug continues to rise when accounting for drug development failures and capital costs. Moreover, it typically takes 5–20 years to create a novel drug due to the extensive preclinical and clinical phases required to prove its efficacy and safety [10]. As a result, drug repurposing has emerged as a promising strategy to more rapidly treat specific diseases, bypassing much of the lengthy and costly drug development process [11]. An outstanding example of drug repurposing occurred during the COVID-19 pandemic, when Remdesivir and Favipiravir, initially approved to treat Ebola and Influenza, respectively, were used at specific stages of COVID-19 treatment [12].

In recent years, computational methods have been pivotal in addressing challenges in precision medicine and drug repurposing. Advancements in statistical learning algorithms, the availability of powerful computing resources, and the accumulation of drug response data are three main factors driving progress in this field. With the advent of free, open-source platforms such as PyTorch and TensorFlow, there has been a significant increase in publications in this field, paving the way for researchers to develop machine learning and deep learning methods to predict drug responses in specific cancer models, most commonly cell lines. Such prediction is central to both drug repurposing and precision medicine. Precision medicine aims to propose the most effective drug(s) among all known treatments. Meanwhile, it is also the ultimate goal of drug repurposing to predict how drugs will respond in a new disease setting [13,14].

The task of drug response prediction can be framed as either a regression or a classification task. Regression is used to predict the continuous value of response representations, such as Area Under the Curve (AUC), Area Above the Curve (AAC), or half-maximal Inhibitory Concentration (IC50) of the dose-response curve. However, in classification tasks, a threshold is used to categorize responses as ‘sensitive’ or ‘resistant,’ or to divide them into multiple classes. Then, a model is developed to classify each drug’s response in the cell line [13].

Another way to define the task is to prioritize and select a list of drugs based on the given cell line features. Learning to Rank (LeToR) is an alternative approach that has received less attention in this field than regression and classification. Ranking can be a more suitable approach because it aligns with the primary goal of drug response prediction models: to propose a list of drugs for clinical users and assist in therapeutic decision-making. Secondly, in vitro experiments that generate drug response data are inherently noisy. Many argue that AUC and AAC are better response representations, as they are more robust than IC50 [15,16]. However, a more effective way to address this issue is to treat AUC or AAC as a comparative score for different drugs in a given cell line, essentially defining a ranking task [13]. In prior comparative studies, listwise LeToR approaches have demonstrated superior performance over pointwise regression models when evaluated using ranking-based metrics, further supporting the suitability of this formulation for drug prioritization tasks. Thirdly, in a ranking task, models simultaneously consider a group of drugs for each cell line, rather than a single drug at a time, as in regression or classification tasks. This approach may also improve model robustness by allowing the system to learn the relative drug ordering.

In recent years, diverse approaches such as Recommender Systems (RS), LeToR, and Reinforcement Learning (RL) have been employed for drug response prediction. RL-based models like Q-Rank and PRORank, introduced in 2015 and 2022, respectively, utilize a Markov Decision Process (MDP) to learn optimal drug ranking policies, with Q-Rank identifying top-performing predictive algorithms and PRORank leveraging Proximal Policy Optimization (PPO) for improved drug recommendations over time [17,18]. RS-based methods like CaDRReS and SRDFM use Matrix Factorization, while SRDFM also incorporates a Siamese Network to evaluate drug relevance per cell line [19,20]. Kernelized Rank Learning (KRL) introduced a kernelized objective to address sparse drug response data and remained a state-of-the-art method for several years after its 2018 release [21]. LeToR techniques vary in complexity: pointwise methods treat ranking as independent regression tasks, pairwise methods, such as pLETORg, optimize the relative ordering of drug pairs [22], and listwise approaches consider the entire drug list, often achieving superior performance [23]. Recent listwise models include ITNR, which employs a Transformer-based architecture to model functional dependencies between genomic features and drugs [24], and an NDCG-optimized version of PaccMann, which enhances the original multimodal IC50 prediction model by incorporating a listwise ranking loss [25,26].

A recent comprehensive study applied pairwise and listwise ranking models to two well-known datasets. It employs gene expression profiles as cell line features and Morgan fingerprints as the drug representation, using encoding layers to generate embeddings. These embeddings are then passed to a scoring function that assigns a score to each drug, which is subsequently used to rank them. This architecture is trained with three novel loss functions introduced in the study [27]: one pairwise ranking loss function, Pair-PushC (derived from the pLETORg model), and two listwise loss functions, ListOne and ListAll.

LeToR has seen significant advancements in recent years. Numerous studies have proposed listwise ranking loss functions demonstrating improved performance across various LeToR benchmarks. Among the most effective loss functions are LambdaLoss, LambdaRank, and NeuralNDCG, which have achieved some of the highest scores on standard benchmarking datasets [28–30]. In parallel, the explainability of LeToR models has emerged as an important area of research. Recently, novel methods such as RankingSHAP and RankSHAP have been introduced to provide better interpretability of model outputs [31,32].

Morgan fingerprints are commonly used to represent molecular structures in many drug response prediction tasks. However, other types of molecular fingerprints, such as AtomPair and Layered fingerprints, remain underutilized in this domain, and no comprehensive benchmarking studies are currently available for these alternatives [33–35]. Given the vast space of possible drug representations, combining multiple molecular descriptors has shown potential benefits. For instance, integrating fingerprints that capture structural features with descriptors that describe physicochemical properties has improved predictive performance in some studies [36,37].

In this paper, we have explored additional listwise ranking loss functions that have demonstrated success in other ranking benchmarks, such as LambdaLoss, LambdaRank, and NeuralNDCG. In addition, we experimented with other molecular representations to see if they could improve the baseline results. Specifically, we employed additional fingerprints, such as AtomPair and RDKit-layered fingerprints. We also used descriptors to capture the physicochemical features of the drugs and combined them with fingerprints to incorporate both structural and physicochemical properties simultaneously. Finally, we investigated various LeToR interpretability approaches to explain why the model ranks drugs in a particular way, focusing on important genes in each cell line and relevant molecular substructures.

In summary, this research makes the following key contributions:

- The impact of various listwise ranking loss functions and molecular representations on multiple drug ranking aspects, including ranking quality across different cell lines and the generalizability of models across datasets, was systematically evaluated.
- Ranking-specific explainability methods, including a proposed modified version of RankinSHAP, were incorporated and systematically assessed. In addition, we proposed an ad-hoc interpretability-oriented architecture that integrates attention with biologically informed protein-protein interaction (PPI) network propagation, embedding drug-target and protein interaction knowledge into the ranking framework to enable biological interpretation.

In the following sections, the overall model architecture employed in this study, the different loss functions, and the proposed explainability techniques are explained. The experimental results across different settings using two benchmark datasets, the implications of these results, and the outlined directions for future research are also presented.

## 2 Materials and methods

### 2.1 Baseline model

In this section, we describe the overall architecture of our baseline ranking model, focusing first on the listwise and pairwise ranking approaches. We then explain how drug and cell line representations are constructed and discuss the scoring mechanism.

#### 2.1.1 Overall architecture.

To rank and prioritize drugs for a given cell line, three key steps must be taken: (1) selecting an appropriate method for drug representation, (2) defining a cell line representation strategy, and (3) implementing a scoring function that assigns a score to each drug based on the given cell line.

**Fig 1:**
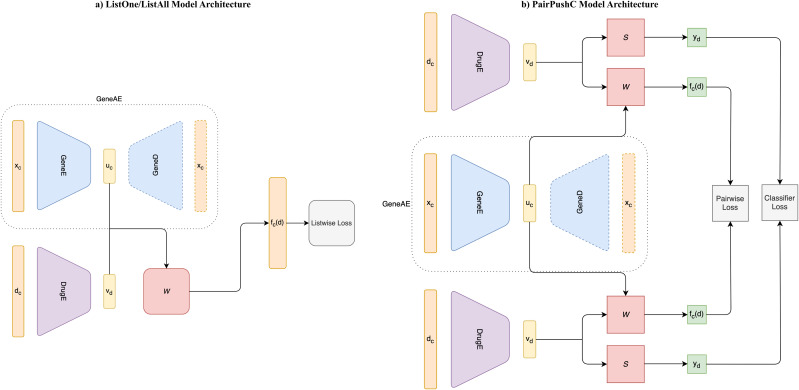
The overall view of model architectures implemented. (a) Listwise Ranking: This architecture updates the DrugE parameters and refines the GeneE encoder within GeneAE while learning a scoring function via W to estimate the correct ordering of drugs for each cell line using listwise loss functions. (b) Pair-PushC: This integrated pairwise ranking model combines drug classification by optimizing DrugE, refining GeneE, and learning a scoring function via W to rank drug pairs while training a classifier via S; the GeneD decoder remains static during training.

Depending on the ranking method employed, the scores assigned to each drug are used differently based on various LeToR methods. In listwise ranking, the model’s output is a list of drug scores, which is then compared to a ground truth list of drug responses, represented as the AUC. This comparison is used to compute the loss and update the model. In contrast, pairwise ranking relies on the relative scores of drug pairs to optimize the model. The loss functions employed for both ranking approaches are discussed in detail in a subsequent section. Fig 1 illustrates the overall architecture of the listwise and pairwise ranking methods.

#### 2.1.2 Cell line representation.

Gene expression profiles are widely used as features to represent cell lines in drug response prediction research [13]. In this study, we input gene expression data from approximately 19,000 genes into our model. Transcriptomics, which involves studying RNA molecules using high-throughput sequencing techniques, enables a comprehensive analysis of both protein-coding and non-protein-coding gene expression [38].

Due to the high dimensionality of gene expression profiles, dimensionality reduction is necessary. In this work, GeneAE was employed, an autoencoder module designed to learn a pre-trained embedding of the cell line representation *u*_*c*_ in a latent space. GeneAE enhances the ranking model by leveraging shared biological features across cell lines and capturing complex, nonlinear interactions among genes.

GeneAE is initially pre-trained to minimize the reconstruction error of gene expression profiles. The loss function used in this module is Mean Squared Error (MSE), and both the encoder (GeneE) and decoder (GeneD) parameters are optimized via backpropagation. This pre-training process is described in Equation 1, where θ is the learnable parameters of the GeneAE and *C* is the set of cell lines. For each dataset and experimental setup, a separate GeneAE model is trained. In the final architecture, the GeneE component is fine-tuned for the ranking task, while GeneD remains frozen.


$$\begin{array}{c} \underset{\theta geneE,\theta GeneD}{\text{min}}\frac{\text{1}}{\left|C\right|}\sum_{c\in C}{\left|\left|x_{c}-\;{x`}_{c}\right|\right|}_{\text{2}}^{\text{2}} \end{array}$$ (1)

#### 2.1.3 Drug representation.

Morgan fingerprint is widely used for describing small molecules in drug response datasets as used in the baseline [27]. In this study, we utilized various molecular fingerprints other than Morgan fingerprint, such as AtomPair and RDKit Layered fingerprints, and a molecular descriptor to represent drug molecules. A detailed explanation of these drug representations is provided in S1.2. The drug representation output consists of a set of features for each drug, which are then passed through DrugE, a multilayer perceptron (MLP), to generate the drug embedding *v*_*d*_. DrugE is trained using backpropagation and is optimized based on the ranking loss function. Depending on the ranking approach, a list or a pair of these embeddings is used to predict the ranking score of each drug for a given cell line.

#### 2.1.4 Scoring function and classifier.

With the drug and cell line embeddings generated by DrugE and GeneE, respectively, a function is required to assign a ranking score, $f_{c}(d)$, to each drug. In our model, the scoring function is defined as a simple weight matrix *W* multiplied by the concatenated embedding vectors of the drug and cell line, as shown in Equation 2. The weight matrix *W* is optimized via backpropagation during training. The final drug scores are sorted to prioritize drugs accordingly.


$$\begin{array}{c} f_{c}(d)=\;u_{c}^{T}Wv_{d} \end{array}$$ (2)

The generated list of scores is sufficient to train the model for listwise ranking. However, in pairwise ranking, an additional binary classifier *S* is introduced to determine whether a drug is sensitive or insensitive. The scoring function also enforces the correct relative ordering of drug pairs. When using Pair-PushC as the primary pairwise ranking loss function, the classifier and the scoring function play essential roles in model optimization (see Fig 1b).

### 2.2 Loss functions

Recent advancements in LeToR tasks have proposed several specialized loss functions for improved convergence and effective ranking of drugs for each cell line. In this section, these loss functions were introduced alongside the three loss functions that form the core of the baseline model.

#### 2.2.1 Pair-PushC loss.

The Pair-PushC loss is derived from the pLETORg model [22]. This loss is designed for pairwise ranking and is combined with a drug classification component. It directly encourages effective drugs to be ranked higher than ineffective ones within each cell line. In doing so, Pair-PushC minimizes the number of cases where an effective drug falls below an ineffective drug while also promoting a correct ordering among the effective drugs themselves. The classification part further helps the model learn discriminative drug embeddings by differentiating between effective and ineffective drugs [27]. The overall objective function for the Pair-PushC loss is defined as follows:


$$\begin{array}{c} \underset{\Theta }{\text{min}}\left\{(\text{1}-\alpha )\;{\text{P}}_{\text{f}}^{↑}\;+\;\alpha \;{\text{O}}_{\text{f}}^{+}\;+\;B_{S}\right\} \end{array}$$ (3)

Where

- Θ denotes all learnable parameters (e.g., embeddings, scoring function weights).
- α is a hyperparameter controlling how much emphasis is placed on the pairwise ordering among effective drugs vs. pushing effective drugs above ineffective ones.
- ${\text{P}}_{\text{f}}^{↑}$, defined in S7, pushes effective drugs above ineffective ones.
- ${\text{O}}_{\text{f}}^{+}$, defined in S8, helps to refine the order among effective drugs.
- $B_{S}$, defined in S6, represents the classification objective.

#### 2.2.2 ListOne loss.

Unlike pairwise approaches, listwise ranking methods consider the entire ranking list for a cell line. ListOne adopts a listwise approach focusing on the top of the ranking list. It estimates the probability that each drug is the most effective (i.e., should be ranked first) within a cell line. This is done by converting the scores for all drugs into a probability distribution (using a softmax function) and minimizing the cross-entropy between the predicted and the ground-truth top-one probability distributions. Essentially, ListOne is tailored to identify the single most effective drug accurately.


$$\begin{array}{c} L_{L\text{𝒾𝓈𝓉}O\text{𝓃𝑒}}\;=\;-\sum_{\text{c}\in C}\sum_{\text{d}\in {\text{D}}_{\text{c}}}{\text{p}}_{\text{c}}^{\text{top}}(\text{d})\;\text{log}\left({\text{p}}_{\text{c}}^{\text{f}}(\text{d})\right) \end{array}$$ (4)

Where:

- $p_{c}^{\text{top}}(d)$ is the ground-truth probability of the drug d being the top-most effective drug in the cell line c.
- $p_{c}^{f}(d)$ is the predicted top-one probability for the drug d in a cell line c.

#### 2.2.3 ListAll loss.

Unlike ListOne, ListAll is designed to consider all effective drugs rather than just the top one. It again uses a parameterized softmax to compute the probability of each drug being effective based on the scores of all drugs in the cell line. The loss function then minimizes the cross-entropy between these predicted probabilities and the actual binary efficacy labels for all drugs. This approach prioritizes every effective drug in the ranking list, making it especially useful when capturing the complete set of promising candidates is important.


$$\begin{array}{c} L_{\text{L\textbackslash{}camtext\{𝒾}}\text{\textbackslash{}camtext\{𝓈}\}\text{\textbackslash{}camtext\{𝓉}\}\text{A\textbackslash{}camtext\{𝓁}\}\text{\textbackslash{}camtext\{𝓁}\}\}\;=\;-\sum_{\text{c}\in C}\sum_{\text{d}\in {\text{D}}_{\text{c}}}{\text{l}}_{\text{c}}(\text{d})\;\text{log}\left({\text{s}}_{\text{c}}^{\text{f}}(\text{d})\right) \end{array}$$ (5)

Where:

- $l_{c}(d)$ is a binary label indicating whether the drug d is effective (1) or not (0) in the cell line c.
- $s_{c}^{f}(d)$ is the score-induced probability that the drug d is effective in the cell line c.

#### 2.2.4 LambdaRank.

LambdaRank [29] modifies the traditional pairwise logistic loss (as used in RankNet [39]) by weighing each document pair by the gain in ranking quality (the change in NDCG) that would result from swapping the two documents. In other words, for each pair (i,j) where document *i* is more relevant than the document j (i.e., $y_{i}>y_{j}$), the loss is the standard logistic loss multiplied by a factor ΔNDCG(i,j) that reflects the impact of their misordering.


$$\begin{array}{c} \text{l}(\text{y},\;\text{s})=\sum_{{\text{y}}_{\text{i}}\;>\;{\text{y}}_{\text{j}}}\Delta \text{NDCG}(\text{i},\text{j}){\text{log}}_{\text{2}}\left(\text{1}+{\text{e}}^{-\sigma \;\left({\text{s}}_{\text{i}}\;-\;{\text{s}}_{\text{j}}\right)}\right) \end{array}$$ (6)

Where:

- $s_{i}$ and $s_{j}$ are the model’s predicted scores for documents i and j,
- σ is a scaling hyperparameter (often set to 1 or tuned),
- ΔNDCG(i,j) is given by the equation below:

$$\begin{array}{c} \Delta \text{NDCG}(\text{i},\text{j})=\left|{\text{G}}_{\text{i}}-{\text{G}}_{\text{j}}\;\right|\times \left|\frac{\text{1}}{{\text{D}}_{\text{i}}}-\frac{\text{1}}{{\text{D}}_{\text{j}}}\;\right| \end{array}$$ (7)

#### 2.2.5 LambdaLoss.

LambdaLoss [30] extends the ideas behind LambdaRank by incorporating a ranking metric (such as NDCG) into the optimization process. In doing so, LambdaLoss leverages a probabilistic framework that defines the ranking loss via the negative log-likelihood of the observed relevance labels given the scores, while marginalizing over all possible ranked lists. Formally, if we let π denote a ranked list (a permutation of the documents) and assume that the model defines a distribution P(π|s) over rankings based on the scores s, then the overall loss is defined as the negative log likelihood:


$$\begin{array}{c} \text{l}(\text{y},\text{s})\;=\;-{\text{log}}_{\text{2}}\text{P}\left(\text{y}|\text{s}\right)\;=\;-{\text{log}}_{\text{2}}\sum_{\pi \in \Pi }\text{P}\left(\text{y}|\text{s},\pi \right)\;\text{P}\left(\pi |\text{s}\right) \end{array}$$ (8)

Where

- y represents the vector of relevance labels,
- s is the vector of scores, and
- Π is a set of all possible ranked lists or permutations
- P(y∣s,π) is the likelihood of observing the labels y given a ranked list π (often defined using a generalized Bradley-Terry model [40] that can be made rank-sensitive).

#### 2.2.6 NeuralNDCG.

NeuralNDCG [28] aims to approximate the standard NDCG metric in a differentiable manner by relaxing the sorting operation, called NeuralSort [41]. This approach uses a smooth approximation of the permutation matrix, denoted as $\hat{P}(s,\tau )$, where τ is a temperature parameter controlling the trade-off between approximation accuracy and gradient smoothness. To further stabilize the approximation, Sinkhorn scaling [42] is applied to ensure that both the rows and columns sum to one.

Once the scaled approximate permutation matrix is computed, the ground truth gains g(y) were sored by left-multiplying them with this matrix. Finally, NeuralNDCG computes a discounted cumulative gain (DCG) from these quasi-sorted gains and normalizes it by the ideal DCG (IDCG) to obtain a differentiable approximation of NDCG@k.

This formulation allows direct optimization of NDCG via gradient-based methods, effectively bridging the gap between the non-differentiable ranking metric and standard LeToR losses. The main formulation (often referred to as NeuralNDCG) is given by:


$$\begin{array}{c} {\text{NeuralNDCG}}_{\text{k}}(\tau )(\text{s},\text{y})\;=\;\frac{\text{1}}{{\text{IDCG}}_{\text{k}}}\;\sum_{\text{j}=\text{1}}^{\text{k}}{\left(\text{scale}\left(\hat{\text{P}}(\text{s},\tau )\right)\cdot \text{g}(\text{y})\right)}_{\text{j}}\text{d}(\text{j}), \end{array}$$ (9)

Where:

- s is the vector of predicted scores,
- y is the vector of ground truth relevance labels,
- g(y) is the gain function applied elementwise on y (typically, $g\left(y_{i}\right)={\text{2}}^{y_{i}}-\text{1}$),
- d(j) is the discount function at rank j (commonly $d(j)=\frac{\text{1}}{{\text{log}}_{\text{2}}(\text{1}+j)}$),
- $\hat{P}(s,\tau )$ is the differentiable approximation to the permutation matrix induced by s (via NeuralSort),
- $\text{scale}\left(\hat{P}(s,\tau )\right)$ denotes the result of applying Sinkhorn scaling to $\hat{P}(s,\tau )$ so that columns sum to one.
- IDCG is the ideal DCG (i.e., maximum possible DCG) computed using ground truth relevance labels sorted in descending order.
- The summation runs over the top *k* ranks.

### 2.3 Explainability methods

To better understand the decision-making processes of the trained models and assess their interpretability, two explainability methods are employed: an attention mechanism [43] and RankingSHAP [31]. These approaches validate the model’s predictions and provide valuable insights into the underlying biological processes and rationale behind the model’s decisions. In the subsequent sections, these two methods are described in detail.

Furthermore, due to the high dimensionality of gene expression data, incorporating prior biological knowledge is essential to prevent overfitting and enhance model relevance. A subset of genes is selected based on biological prior knowledge described in S1.4 in S1 File to address these challenges. This technique leverages known PPI and drug-target interactions to refine and prioritize gene expression profiles. In practice, it involves propagating signals across a PPI network to identify genes that are highly expressed and central to key biological pathways. Following the methodologies outlined in S1.4 in S1 File, this approach filters out genes with low or redundant biological significance, effectively reducing model complexity. The result is a more focused set of features, consisting of 2882 for CTRP and 4899 for PRISM out of 19177 gene-expression profiles for each cell line, better capturing the underlying biology. This methodology ultimately improves both the interpretability and predictive performance of the model.

#### 2.3.1 Attention mechanism integration.

An attention mechanism (explained in S1.3 in S1 File) was integrated into the baseline model architecture to further enhance interpretability and performance. This module is designed to weigh the different inputs dynamically, effectively allowing the model to “focus” on the most relevant features when computing the final ranking scores. During training, the attention mechanism learns which parts of the input data (for example, certain genomic features) are most indicative of drug efficacy. The attention weights can be visualized or extracted post-training, offering a transparent view of the decision process. This mechanism serves a dual purpose: it improves the model’s accuracy by emphasizing important features and makes ranking decisions more interpretable.

**Fig 2:**
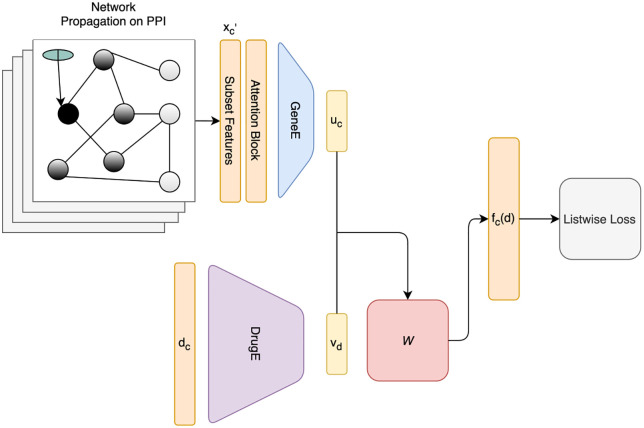
The figure presents the proposed architecture of using an attention mechanism to demystify the model’s ranking procedure. The gene features are first refined through network propagation on a PPI network, followed by subset selection and an attention block. GeneE encodes these processed features into a latent representation. In parallel, drug features are encoded by DrugE. The resulting gene and drug embeddings are combined via a learnable scoring module W to produce effective scores. These scores are optimized using a listwise loss function, enabling the model to rank drugs effectively for each cell line.

A self-attention block was embedded after the cell-line input, following a linear layer with a ReLU activation function to encode the features into a lower-dimensional space for the scoring function. The rest of the architecture remained as described in Section 2.1. The complete architecture is shown in Fig 2.

#### 2.3.2 RankingSHAP.

Shapley values have been widely used for explaining machine learning models by attributing the contribution of each feature to a model’s prediction in a fair and theoretically grounded manner [44]. However, traditional SHAP-based methods struggle when applied to ranking models, as they fail to capture the listwise dependencies inherent in ranking tasks [45]. To address this limitation, RankingSHAP has been proposed as a listwise feature attribution method that extends SHAP to ranking models by considering the order of ranked items in its explanations [31].

RankingSHAP explains the features of drugs in our model. It applies a listwise mask to all drugs in a ranking, isolating the contributions of non-masked features. It then reduces the model’s predictions to a single value, like Kendall’s tau, to measure how feature perturbations impact the ranking, identifying key features that influence the overall order.

Additionally, we introduced an innovation based on RankingSHAP to explain the gene expressions of cell lines in our model. The model takes a single cell line and ranks all the drugs in the dataset. At each stage, certain gene expressions of the cell line are masked to assess their impact on the ranking decision. How their absence affects the model’s predictions can be analyzed by disregarding specific gene expressions. Similar to RankingSHAP, the predictions are then reduced to a single value, allowing us to quantify how feature perturbations influence the ranking and identify key features that shape the overall order.

A ranking model was considered:


$$\begin{array}{c} R:\left\{V_{c}\;,D_{d}\right\}\to \pi_{c} \end{array}$$ (10)

Which maps a gene expression profile $V_{c}$ of a given cell line c and a set of candidate drug feature vectors $D_{d}={\left\{d_{j}\right\}}_{j}$ to a permutation matrix $\pi_{c}$ that represents the ranked list of drugs.

In practice, a model is trained using a listwise loss to predict a ranking score for each drug $d_{j}$ based on how well it matches the gene expression profile of the given cell line. The ranking process follows:


$$\begin{array}{c} \tilde{R}:D\to R,\;d_{j}\mapsto s_{c,j} \end{array}$$ (11)

where $\tilde{R}$ assigns a score $s_{c,j}$ to each drug $d_{j}$. The final ranked list is obtained by sorting these scores:


$$\begin{array}{c} R=ranked\circ \prod_{\left|D_{d}\right|}\tilde{R} \end{array}$$ (12)

This ranking model helps identify the most relevant drugs for a given cell line based on gene expression, aiding in drug repurposing and personalized medicine.

**Algorithm 1**: adjusted model prediction (used in combination with SHAP)

**Require**: Ranking model R, feature vectors $D_{d}$ for drug d, listwise explanation g

**Input**: masking function $m_{t,b}$, gene expression $V_{c}$ for cell-line

1 ${\tilde{V}}_{c}\leftarrow m_{t,b}$($V_{c})$

2 $\pi \leftarrow R({\tilde{V}}_{c},\;D_{d}\;)$

Initialize the inner loop.

3 υ←g(π)

repeat

4 return υ

 $z^{n,t+\text{1}},{\hat{z}}^{n,t+\text{1}}\leftarrow PrimalDualStep\left(z^{n,t},\eta \right)$

In the following section, experimental settings, datasets used in this study, and the results obtained from applying the proposed loss functions and molecular representations are presented. Additionally, the outcomes of the novel explainability methods introduced earlier are reported.

### 2.4 Ethics statement

This study exclusively used publicly available, de-identified datasets and did not involve human participants, animal experiments, or access to identifiable personal data. Therefore, ethics approval and informed consent were not required.

## 3 Experiments

### 3.1 Experiment setting

The experiments’ settings are identical to [27]. The following defines each part of the settings, such as data preparation, training, validation setups, and performance evaluation, in detail.

#### 3.1.1 Data experiment settings.

According to [27], the dataset setting is the same regarding data sampling and experimental setups. This study collected and prepared data from two large-scale public datasets. The datasets, CTRP (Cancer Therapeutic Response Portal) and PRISM, comprise a high number of cancer cell lines along with drug response measurements. Drug response values are quantified as the AUC, with lower values indicating higher drug sensitivity. Gene expression data were obtained from the Cancer Cell Line Encyclopedia (CCLE) and used for pretraining a cell line encoder (GeneAE) that extracts latent representations. Similarly, drug molecular fingerprints (Morgan-Count with 2048, RDKit-layered with 200, and AtomPair with 1024 dimensions) and RDKit descriptor (with 210 dimensions) were used to learn drug embeddings via a dedicated neural network module (DrugE). These embeddings serve as the foundation for the subsequent ranking tasks.

#### 3.1.2 Train-validation setups.

The experimental evaluation is conducted under two primary settings, each designed to reflect a different real-world scenario. In the first setting, termed the “leave-cell lines-out” (LCO) validation, cell lines are partitioned by cancer type into five folds. In each iteration, three folds are used for training, one for hyperparameter tuning, and the remaining fold is held out as unseen data for testing. To ensure methodological rigor and prevent information leakage, the GeneAE model is pretrained independently for each fold. Specifically, the GeneAE encoder for a given fold is trained exclusively on the genomic profiles of the cell lines within that fold’s training and validation sets, ensuring that cell lines in the test set remain entirely unseen during both pretraining and downstream prediction. This setup simulates a situation in which drugs are ranked for new, previously unseen cell lines, with the cell line embeddings for the test set being obtained by interpolation from nearby training cell lines in the latent space. In the second setting, referred to as “leave-responses-out” (LRO) validation, the entire set of cell lines is known, but for some cell lines, drug response values for a subset of drugs are intentionally held out. This configuration mimics a drug repurposing scenario where known drugs are tested in additional cell lines, ensuring that each drug is paired with at least one cell line and each cell line is paired with at least one drug in the training data [27].

For the training setting, the Adam optimizer was employed for all training runs, setting an initial learning rate of 1e-3 based on preliminary tuning experiments. Each fold in our cross-validation setup was trained for 100 epochs, ensuring consistent training across all folds. These hyperparameters and additional settings, such as batch size and regularization factors, were fine-tuned on the validation sets. The codes were implemented using PyTorch in Python, and the experiments were conducted on three main servers equipped with two NVIDIA RTX 3090 Ti GPUs and one NVIDIA RTX 4090 GPU.

#### 3.1.3 Performance evaluation.

Evaluation of the models is based on several ranking metrics, which is introduced in detail in Section S1.1 in S1 File. The primary metrics include the average precision at K (AP@K) and the hit rate at K (AH@K), which measure the accuracy of the top-K predictions and the number of truly effective drugs ranked within the top K positions. In addition, the overall ranking quality is assessed using the Concordance Index (CI) [5]. While preserving the overall ranking structure is important, the primary interest is in accurately prioritizing the top effective drugs; hence, NDCG@K [6] is prioritized when comparing model performance.

### 3.2 Explainability setting

Additionally, explainability mechanisms were incorporated to assess performance and provide insight into the models. An attention mechanism [43] was integrated within the architecture to highlight key signals influencing the ranking decisions. In conjunction, PPI network [46] propagation was utilized to extract and prioritize gene expression profiles of interest, following the setups described in [25,26], which not only enhanced biological relevance but also reduced model complexity by filtering out less informative genes. The details of this gene selection method are provided in S1.4 in S1 File. The details of the explainability alongside its results will be broken down in the following sections. Furthermore, RankingSHAP [31] quantified the importance of features in the outputs. To reduce memory consumption during the calculation of SHAP values for gene expression, the list of genes mentioned in S1.4 in S1 File was selected, and Kernel SHAP was applied to these selected genes. Due to limited memory and computational resources, the number of background samples for genes and drugs was set to 4 and 2, respectively.

### 3.3 Datasets

In this research, we utilize two datasets widely recognized as the most appropriate for training and evaluating drug response prediction models. Their strengths lie in the diversity of experiments, testing various drugs on multiple cell lines, and their complementary coverage of both drugs and cell lines.

The first dataset is the CTRP, published by the Broad Institute. The version released in 2015 reports experimental results from studies on standard cancer cell lines treated with small-molecule drugs, aiming to accelerate precision medicine [47,48].

The second dataset is based on the PRISM drug repurposing project, available through the DepMap Portal and first compiled in 2019. This dataset was generated by testing numerous small-molecule drugs on a wide array of cell lines using the Profiling Relative Inhibition Simultaneously in Mixture (PRISM) method, a molecular barcoding approach that allows drugs to be screened in pooled cell line experiments. The high-throughput nature of this method yields a broader spectrum of drug data [49].

To obtain omics data for the cell lines present in both datasets, we leveraged the CCLE [48]. A significant subset of the cell lines in CTRP and PRISM overlap with those in CCLE. We used gene expression data for model training to represent cell line characteristics, while mutation data was incorporated for additional model interpretability.

For consistency and improved generalizability, we employed the adjusted AUC as our response metric [15,16,50]. Notably, 10.2% and 18.9% of responses were missing for PRISM and CTRP, respectively. Because the task is a ranking problem, pairs of drugs and cell lines with missing response values were excluded from model training. Similarly, cell lines lacking gene expression data in CCLE were removed from the analysis.

**Fig 3:**
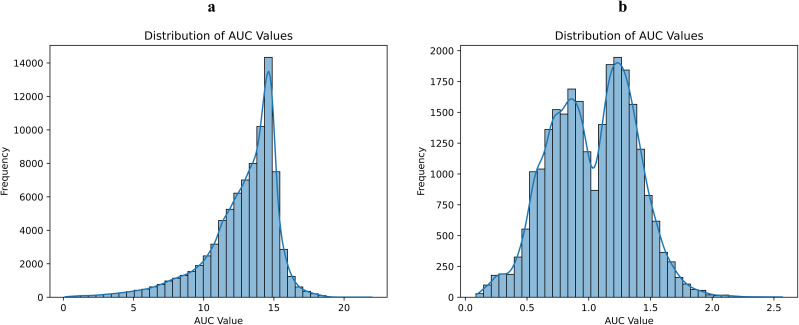
The figures show the distribution of responses in the CTRP (a) and PRISM (b) datasets.

S1 Table in S1 File summarizes the key features of the two datasets, while Fig 3 illustrates the distribution of AUC responses across the datasets. The difference between the distribution of data in the two datasets is due to the differences in the response assay and the preprocessing of AUCs [16]. In CTRP, AUCs were recalculated with a standard concentration range to compare responses within the dataset, while in PRISM, AUCs were calculated using the 8-point concentration across the cell line-drug pair [27].

### 3.4 Hyperparameter sensitivity and optimization analysis

To ensure a fair and rigorous comparison between the ranking loss functions, a standardized hyperparameter optimization (HPO) and sensitivity analysis protocol was implemented. For baseline models, the optimal configurations reported in their respective original studies [27] were adopted to ensure evaluation at maximum potential capacity. For the other loss functions, including NeuralNDCG, LambdaLoss, and LambdaRank, a grid-based HPO was conducted using a minimal and consistent training configuration under the LCO setup and CTRP dataset. Additionally, for comparison purposes, NDCG and AP@10–20 were prioritized over global metrics to focus on the high-precision retrieval of top lead compounds. This threshold provides a meaningful benchmark for identifying the most potent drug-cell line interactions within a vast search space.

The sensitivity of the models was investigated across several key parameters, including the scaling factors μ and σ for LambdaLoss and LambdaRank, the temperature parameter for NeuralNDCG, α and β for PairPushC, and the list-size parameter *m* for ListOne and ListAll. As illustrated in the sensitivity maps provided in the S1-S6 Figs in S1 File, the framework demonstrates a high degree of stability across a wide range of parameterizations.

Specifically, for the LambdaLoss function, performance variance remained remarkably low, with a maximum delta (max/min difference) in NDCG@10–20 of only 0.0051 and a delta in AP@10–20 of 0.0263 across the search space. Similarly, LambdaRank exhibited a delta of 0.0083 for NDCG@10–20. While the ListOne and ListAll methods showed higher sensitivity to the list-size parameter *m* (with deltas of 0.2038 and 0.4069, respectively), the overall performance across the proposed methods remained competitive with or superior to the baselines.

### 3.5 Overview of ranking loss functions and molecule representations

All combinations of the six ranking loss functions and five molecular representations were systematically evaluated across datasets and validation setups. Given the large number of performance metrics, models were first ranked per metric using the Friedman procedure, and average ranks were computed to enable an overall comparison. To determine whether observed performance differences were statistically significant, the Friedman test was formally applied, followed by the Nemenyi post-hoc test for pairwise comparisons (Friedman Chi-Squared = 2.9861, p-value = 0.99). When considering the complete set of evaluation metrics jointly, the statistical analysis indicated that no single combination of loss function and molecular representation consistently outperformed all others, suggesting the absence of a universally optimal configuration. However, aggregating all ranking metrics into a single global comparison may mask meaningful distinctions, as different metrics capture different aspects of ranking quality, e.g., early retrieval versus overall ordering consistency. Therefore, additional analyses were conducted on more specific and practically relevant subsets of metrics. Applying the Friedman and Nemenyi tests to these targeted metric groups provided more informative insights into model behavior. The overall Friedman rankings and the metric-specific results are presented in the following sections, with complete tables reported in the Supporting Information.

**Fig 4:**
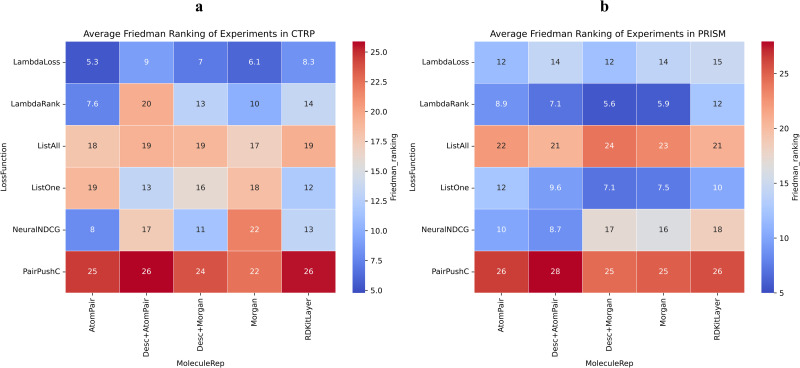
The plots illustrate the average Friedman ranking of all performance metrics for the experiments in the CTRP (a) and PRISM (b) datasets for the LCO setup.

The plots in Fig 4 demonstrate that the choice of loss function influences model performance in the LCO setting. In the CTRP dataset, LambdaLoss achieved the lowest Friedman ranking, indicating superior performance, while Pair-PushC performed the worst for training a ranking model on drug response data. In contrast, LambdaRank consistently performed best across its various combinations with different molecule representations in the PRISM dataset, whereas Pair-PushC yielded the poorest results again.

For the CTRP dataset, the AtomPair representation proved to be the most effective when used with our newly evaluated loss functions (LambdaLoss, LambdaRank, and NeuralNDCG). Conversely, for the baseline loss functions (ListAll, ListOne, and Pair-PushC), the Morgan fingerprint, and its combination with a molecular descriptor, delivered the best performance. The Morgan fingerprint (with and without a molecular descriptor) performed better in the PRISM dataset when used with LambdaRank, ListOne, and Pair-PushC. In contrast, the AtomPair representation (with and without a molecular descriptor) showed only marginal improvements in other experiments.

In the LRO setting, LambdaLoss significantly outperformed the other loss functions on the CTRP dataset, particularly when combined with the AtomPair fingerprint with a molecular descriptor (see S7a Fig in S1 File). In PRISM, LambdaLoss and LambdaRank produced superior average results across different molecule representations; however, ListOne combined with the Morgan fingerprint yielded the lowest Friedman ranking among all experiments (see S7b Fig in S1 File).

Based on the Friedman ranking, the best model for the CTRP dataset in the LCO setup is the combination of LambdaLoss with the AtomPair fingerprint. LambdaRank with the Morgan fingerprint combined with a molecular descriptor in the PRISM dataset performs best.

### 3.6 The metric-wise results of models

This section evaluates various metrics at multiple Ks (1, 3, 5, 10, 20, 40, 60) across both datasets and evaluation scenarios (LCO and LRO) for different combinations of loss functions and molecule representations. The AH metric was rescaled by dividing its values by the maximum value to illustrate the plots better. Additionally, average results were calculated across loss functions and molecular representations for each combination of setup and dataset. Initially, the loss functions were investigated, followed by examining the influence of molecular representations. Also, the result of Friedman and Nemenyi tests for metrics in various values of Ks are shown in S8-S19 Figs in S1 File.

#### 3.6.1 Comparison based on loss functions.

**Fig 5:**
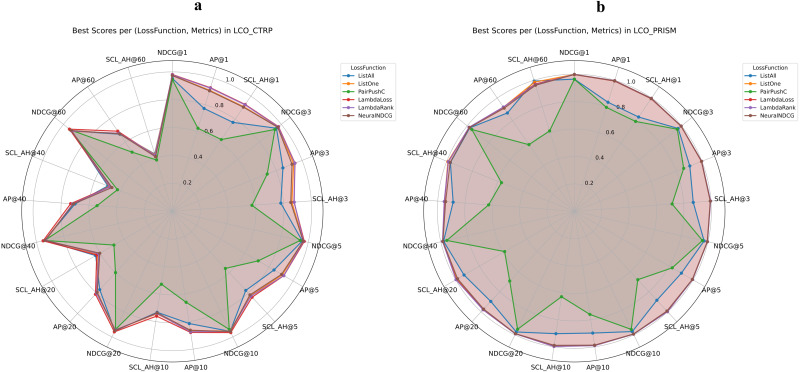
The spider-plots of evaluation metrics at different Ks for the CTRP (a) and PRISM (b) datasets in the LCO setup across all ranking loss functions. The “SCL” prefix of AH is the short form of the word “Scaled”.

Fig 5 and S20 Fig in S1 File show that the larger the area covered by a loss function, the better its overall performance. According to the plots across all scenarios and datasets, LambdaLoss exhibits the best performance, closely followed by LambdaRank and ListOne, with only marginal differences. NeuralNDCG ranks second and shows intermediate performance across most setups, except in the LRO scenario with the CTRP dataset, where it demonstrates the lowest performance compared to other loss functions. The remaining loss functions, including ListAll and Pair-PushC, consistently show lower performance, with Pair-PushC at the bottom of the ranking.

Another interesting observation across performance metrics is that all loss functions exhibit nearly equivalent performance in terms of NDCG. However, Pair-PushC notably outperforms other loss functions, specifically in the LRO scenario for the CTRP dataset, indicating its superior capability in effectively ranking the model.

From another perspective, within the CTRP dataset under the LCO scenario, certain loss functions consistently outperform others, particularly at lower Ks (1, 3, and 5). The differences between loss functions decrease as Ks increase, suggesting that higher Ks may normalize performance disparities. Conversely, the PRISM dataset reveals more pronounced distinctions among loss functions at intermediate Ks (10, 20), highlighting differing optimization focuses of these loss functions.

The LRO scenario provides an additional viewpoint, showing more pronounced variations in loss function performance across the two datasets. Specifically, CTRP demonstrates robust performance for certain loss functions (LambdaLoss, LambdaRank, and ListOne) at smaller Ks, indicating stronger predictive capabilities at early retrieval stages. Similarly, the PRISM dataset shows distinct strengths for specific loss functions (LambdaLoss, LambdaRank, ListOne, and NeuralNDCG) at intermediate and higher Ks, underscoring that dataset-specific characteristics significantly impact the efficacy of loss functions.

#### 3.6.2 Comparison based on molecule representations.

**Fig 6:**
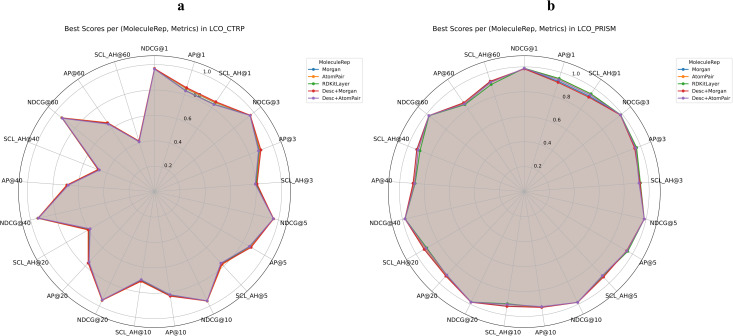
The spider-plots of evaluation metrics at different Ks for the CTRP (a) and PRISM (b) datasets in the LCO setup across all molecule representations. The “SCL” prefix of AH is the short form of the word “Scaled”.

According to Fig 6, overall, there is no significant difference in performance across the molecule representations. Additionally, the behavior of the models in various Ks remains consistent in different setups, as discussed in the loss functions section. As the value of k increases, the performance of the models generally declines. Except for the PRISM dataset under the LCO evaluation setup, the trend in Ks does not significantly affect the models’ performance.

The LRO scenario exhibits a similar pattern, showing the least distinct performance variations among molecular representations. Specifically, in the CTRP dataset, all representations consistently perform better at lower Ks than higher Ks. In the PRISM dataset, similar trends are observed across different K ranges as in CTRP, although the metric values at higher Ks are better than those in CTRP. The corresponding plot for the LRO setups can be seen in S21 Fig in S1 File.

### 3.7 Explicit evaluation of ranking loss functions under varying degrees of data imbalance

To explicitly assess the robustness of different ranking loss functions to dataset imbalance, we conducted controlled experiments in which varying degrees of imbalance between effective and ineffective drugs were artificially induced for each cell line. The objective was to evaluate how well each loss function maintains ranking performance when the proportion of sensitive and insensitive drugs changes.

Imbalance was introduced through a biased sampling strategy that selectively sampled drugs along the response spectrum, from the least effective to the most effective. The degree of imbalance was controlled by a parameter 𝛼 ∈ [−1,1]. When 𝛼 = 0, the sampled drug set is balanced across effectiveness levels. Negative values of 𝛼 bias the sampling toward ineffective drugs, with 𝛼 = −1 corresponding to a set composed entirely of the least effective drugs. Conversely, positive values of 𝛼 bias the sampling toward effective drugs, with 𝛼 = 1 representing a set dominated by the most effective drugs.

The ranking performance of each loss function was evaluated using NDCG at multiple cutoff levels (grouped as @1,3,5; @10,20; and @40,60) across the full range of 𝛼 values. As illustrated in S22 Fig in S1 File, the performance of all evaluated loss functions remained relatively stable across different imbalance levels, indicating that the ranking losses demonstrate robustness to substantial shifts in the distribution of effective versus ineffective drugs.

### 3.8 Generalizability

The three best models for each setup and dataset pair were selected based on the Friedman test ranking to assess model performance on unseen datasets. Then, each model’s performance was evaluated on the counter dataset using the same ranking methods to determine which models were the most generalizable overall.

Based on the results in Section 3.3, the top three models for the LCO setup on the CTRP dataset use the LambdaLoss loss function with AtomPair, Morgan, and Descriptor + Morgan molecule representations. In contrast, the PRISM dataset’s best models employ the LambdaRank loss function with Descriptor + Morgan, Morgan, and ListOne loss functions with Descriptor + Morgan, respectively. For the LRO scenario, the optimal CTRP models combine LambdaLoss with Descriptor + AtomPair, Descriptor + Morgan, and LayeredRDKit. In contrast, in PRISM, the best-performing models utilize the ListOne loss function with Morgan and the LambdaRank loss function with LayeredRDKit and Descriptor + Morgan representations.

Table 1, corresponding to the LCO setup, is divided into two subsets based on the dataset used. For the CTRP dataset, three models are evaluated using the LambdaLoss function with different molecular representations: AtomPair, Morgan, and Desc+Morgan. The results indicate that while the overall ranking quality, measured by the CI, is similar across these models (ranging from 0.597 to 0.606), the AtomPair representation yields superior early ranking performance. This is evident from its higher AP and AH scores. Although the AtomPair representation also performs better in terms of NDCG, at higher Ks (NDCG@40–60), the combination of descriptors and the Morgan fingerprint marginally outperforms the other models.

**Table 1:** Models’ generalizability performance in the LCO setup. Highlighted values indicate the best performances. The first three rows are the models trained on CTRP and evaluated on the PRISM dataset. On the other hand, the second three rows are models trained on the PRISM dataset and evaluated on CTRP.

Loss Function	Molecule Representation	CI	AP 1-3-5	AP 10-20	AP 40-60	AH 1-3-5	AH 10-20	AH 40-60	NDCG 1-3-5	NDCG 10-20	NDCG 40-60
LambdaLoss	AtomPair	0.597	**0.564**	**0.536**	**0.447**	**1.316**	**6.139**	**14.878**	**0.921**	**0.908**	0.860
LambdaLoss	Morgan	0.605	0.529	0.496	0.404	1.167	5.283	14.282	0.911	0.892	0.875
LambdaLoss	Desc+Morgan	0.606	0.528	0.506	0.419	1.205	5.606	14.476	0.914	0.901	**0.881**
LambdaRank	Desc+Morgan	0.630	0.178	0.274	0.293	0.414	3.584	12.985	0.769	0.782	0.815
LambdaRank	Morgan	**0.632**	0.148	0.264	0.282	0.412	3.357	12.956	0.724	0.749	0.802
ListOne	Desc+Morgan	0.629	0.146	0.242	0.262	0.346	3.039	12.426	0.663	0.702	0.769

In contrast, the PRISM subset within the LCO setup employs ranking-based loss functions, LambdaRank and ListOne, paired with Morgan and Desc+Morgan representations. Although these models achieve higher CI values (ranging from 0.629 to 0.632), they exhibit a noticeable drop in early ranking metrics, with lower AP and AH scores compared to their CTRP counterparts. In particular, the ListOne model shows the lowest overall performance across all metrics. In general, scores in this subset are lower than those observed in the models trained on CTRP, demonstrating that models trained on the CTRP dataset are more generalizable than those trained on PRISM.

On the other hand, S2 Table, representing the LRO setup, is divided into two dataset-specific segments as well. For the CTRP dataset, three models using LambdaLoss with different molecular representations, Desc+AtomPair, Desc+Morgan, and RDKitLayer, are compared. Here, the Desc+Morgan model achieves the highest CI (0.608), while the Desc+AtomPair representation leads in early ranking performance, as indicated by the highest AP scores. The performance of these two molecule representations is slightly competitive, with Desc+Morgan excelling at higher Ks (@40–60) and Desc+AtomPair performing better at lower Ks (@1-3-5) in both AH and NDCG. The performance trends in the LRO CTRP subset are similar to those observed in the LCO setup. However, the absolute metric values differ slightly, reflecting the sensitivity of model performance to the experimental configuration.

For the PRISM dataset in the LRO setup, three models are trained using ListOne and LambdaRank loss functions combined with Morgan, RDKitLayer, and Desc+Morgan representations. The highest CI in this subset is achieved by the ListOne model with Morgan at 0.630. However, the LambdaRank models outperform in AP, AH, and NDCG scores, indicating a more favorable ranking of the most relevant items despite slightly lower CI values. Specifically, the RDKitLayer model performs marginally better at intermediate Ks (@10–20), while the Desc+Morgan model excels at higher Ks (@40–60) in both AH and NDCG. Overall, the metrics remain lower in this subset, mirroring the pattern seen in the LCO PRISM subset, where the overall ranking order improves, but early retrieval precision diminishes.

Overall, both experimental setups show that models trained on CTRP generally offer higher generalizability (AP, AH, NDCG), whereas the models trained on PRISM deliver superior overall ranking quality (CI). In the LCO setup, LambdaLoss performs best for CTRP, while ranking losses like LambdaRank and ListOne enhance CI for PRISM. The LRO setup mirrors these trends, with a notable trade-off between CI and early ranking metrics across different Ks. This analysis highlights the critical role of selecting the right loss function and molecular representation to meet specific performance goals.

### 3.9 Ablation study of descriptors and fingerprints

To better understand the effect of concatenating descriptors with fingerprints, we selected Morgan and AtomPair as fingerprints and ListAll and LambdaRank as the best-performing baseline and proposed loss functions, respectively. The experiments were conducted on both datasets and across different setups to examine the impact of this concatenation.

From an overall perspective, using descriptors alone yields the lowest values in nearly all metrics across both datasets and setups. However, incorporating descriptors with other fingerprints can improve performance in certain metrics (see Tables 2 and 3).

**Table 2:** The ablation study of fingerprint and descriptor effectiveness in the CTRP dataset and LCO setup.

Descriptor	Morgan	AtomPair	Loss Function	AH 1_3_5	AP 1_3_5	NDCG 1_3_5	AH 10_20	AP 10_20	NDCG 10_20	AH 40_60	AP 40_60	NDCG 40_60	CI
		*	LambdaRank	**2.587**	0.937	0.974	**8.969**	**0.860**	**0.957**	18.188	**0.712**	**0.934**	0.682
		*	ListAll	2.294	0.823	0.958	8.840	0.795	**0.957**	**18.407**	0.685	**0.934**	0.683
*			LambdaRank	0.467	0.315	0.718	1.228	0.323	0.647	5.010	0.200	0.653	0.770
*			ListAll	0.660	0.395	0.788	1.593	0.395	0.616	5.085	0.246	0.628	0.770
*		*	LambdaRank	2.471	0.930	0.971	8.199	0.831	0.944	16.987	0.666	0.915	0.578
*		*	ListAll	2.358	0.840	0.956	8.743	0.809	0.954	18.151	0.684	0.930	0.586
*	*		LambdaRank	2.560	0.932	0.972	8.795	0.852	0.952	18.017	0.701	0.929	**0.771**
*	*		ListAll	2.284	0.794	0.952	8.886	0.794	0.955	18.406	0.686	0.932	0.683
	*		LambdaRank	2.585	**0.938**	**0.975**	8.773	0.858	0.954	17.925	0.704	0.930	0.740
	*		ListAll	2.360	0.841	0.957	8.898	0.812	0.955	18.271	0.693	0.931	0.688

**Table 3:** The ablation study of fingerprint and descriptor effectiveness in the PRISM dataset and LCO setup.

Descriptor	Morgan	AtomPair	Loss Function	AH 1_3_5	AP 1_3_5	NDCG 1_3_5	AH 10_20	AP 10_20	NDCG 10_20	AH 40_60	AP 40_60	NDCG 40_60	CI
		*	LambdaRank	2.974	**0.998**	0.997	12.870	**0.988**	0.994	38.658	**0.938**	**0.985**	0.649
		*	ListAll	2.680	0.914	0.973	11.824	0.912	0.976	39.083	0.879	0.978	0.669
*			LambdaRank	1.778	0.885	0.953	2.707	0.845	0.875	7.192	0.462	0.761	0.816
*			ListAll	1.369	0.568	0.779	3.767	0.560	0.730	10.447	0.375	0.676	0.816
*		*	LambdaRank	**2.976**	**0.998**	**0.998**	12.848	**0.988**	0.994	38.843	0.937	**0.985**	0.606
*		*	ListAll	2.713	0.930	0.974	11.989	0.921	0.978	39.165	0.888	0.978	0.586
*	*		LambdaRank	2.975	**0.998**	**0.998**	12.873	**0.988**	**0.995**	39.258	**0.938**	**0.985**	**0.817**
*	*		ListAll	2.459	0.811	0.960	11.667	0.869	0.973	39.239	0.869	0.978	0.66
	*		LambdaRank	2.975	**0.998**	**0.998**	**12.884**	**0.988**	0.994	39.170	**0.938**	**0.985**	**0.817**
	*		ListAll	2.493	0.838	0.963	11.666	0.874	0.973	**39.291**	0.871	0.978	0.674

In the CTRP dataset under the LCO setup, adding descriptors to the AtomPair fingerprint does not generally enhance the overall model performance. However, at K values below 20, it results in higher AP and AH values when used with ListAll. Additionally, at larger K values, the combination of descriptors with Morgan leads to improvements in AH or NDCG. For example, when using LambdaRank as the loss function, adding descriptors to Morgan increased the average AH at K = 40 and K = 60 from 17.90 to 18.01, and at K = 10 and K = 20 from 8.77 to 8.79.

In the PRISM dataset under the LCO setup, adding descriptors has the most notable effect on the AtomPair fingerprint. Across almost all metrics, including descriptors, improves performance when paired with AtomPair and the ListAll loss function. For models trained with LambdaRank, the values for different molecular representations are relatively close, though minor improvements can be observed with adding descriptors. For instance, adding descriptors increased AH by 0.2 when using AtomPair and Morgan with LambdaRank for Ks = 40 and 60.

In the CTRP dataset under the LRO setup, concatenating descriptors with the AtomPair fingerprint improves performance when LambdaRank is used as the loss function, showing gains across almost all metrics except CI. Similarly, concatenation with Morgan improves performance under ListAll in several metrics, though not universally. However, in contrast to CTRP, adding descriptors to the AtomPair in PRISM does not enhance model performance. Instead, Morgan combined with LambdaRank demonstrates consistent improvements across all metrics (see S3 and S4 Tables in S1 File).

### 3.10 Metric improvement effect on ranking quality of drugs

To evaluate whether improvements in ranking metrics correspond to meaningful gains in practical drug prioritization, a comparative analysis was conducted using the best and worst performing models across each dataset and experimental setup.

Two biologically distinct cancer types with targeted therapies were selected: non-small cell lung cancer (NSCLC) and melanoma. In the evaluated setting, NSCLC is associated with three clinically established *EGFR* inhibitors, Erlotinib, Gefitinib, and Afatinib [51–53]. In contrast, melanoma is characterized by sensitivity to *BRAF/MEK* pathway inhibitors, including Dabrafenib and Trametinib [54]. The clear therapeutic separation between these two diseases enables assessment of whether ranking improvements reflect biologically meaningful drug prioritization rather than random variation.

Cell lines corresponding to NSCLC and melanoma were selected, and each model was tasked with ranking a fixed list of the five aforementioned drugs. The objective was to determine whether clinically relevant drugs were assigned higher ranking positions for their corresponding cancer type. This experimental design enables evaluation of the relationship between metric improvement and disease-specific drug prioritization in a practical and interpretable scenario.

**Fig 7:**
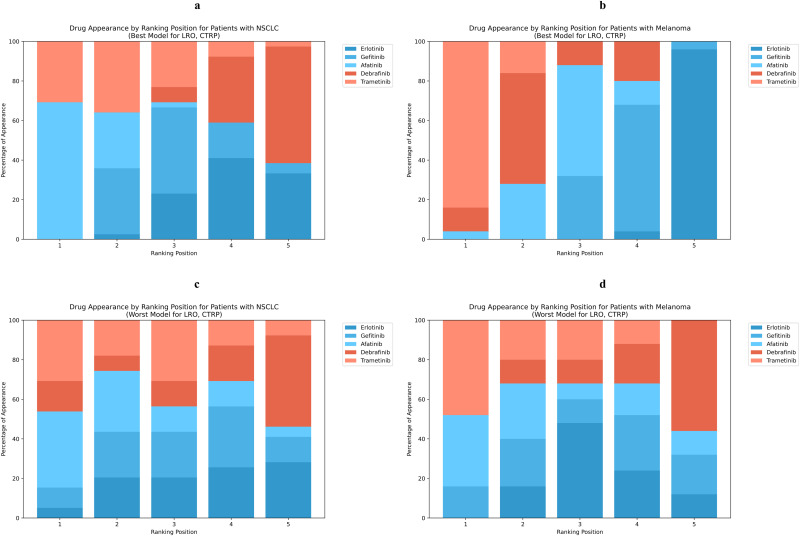
Comparative evaluation of drug ranking behavior in the LRO setup trained on the CTRP dataset. The figure illustrates the prioritization of clinically established drugs for NSCLC and melanoma using the best and worst performing models: (a) Best model for NSCLC, (b) Best model for melanoma, (c) Worst model for NSCLC, and (d) Worst model for melanoma.

Fig 7 illustrates the results obtained from the best-performing model in the LRO setup trained on the CTRP dataset. The model demonstrates consistent prioritization behavior: for NSCLC cell lines, Erlotinib, Gefitinib, and Afatinib predominantly appear within the top three ranking positions, whereas for melanoma cell lines, Dabrafenib and Trametinib are ranked higher. In contrast, the worst performing model exhibits less consistent and more dispersed ranking patterns, with disease-specific drugs appearing across various ranking positions without clear prioritization. Results corresponding to additional setups and datasets are presented in S23-S25 Figs in S1 File.

### 3.11 Explainability of models on breast cancer cell lines

We employed two interpretability approaches to gain a deeper understanding of model behavior: (1) RankingSHAP, a post-hoc SHAP-based method tailored for ranking tasks, and (2) an ad-hoc attention-based model. The two top-performing models in the LCO setting were selected for the RankingSHAP analysis. We retained the molecular representation and loss function from the best LCO models for the attention-based model but modified the architecture to incorporate an attention mechanism. We focused on BC cell lines to evaluate whether these models can identify biologically and chemically relevant features of both the cell lines (e.g., key gene markers) and the drugs (e.g., important chemical substructures). BC was selected because it is one of the most molecularly characterized cancers, with well-established and biologically distinct subtypes such as ER-positive and ER-negative disease, providing a reliable and mechanistically grounded framework for validating subtype-specific interpretability findings.

#### 3.11.1 Cell line explainability.

**Fig 8:**
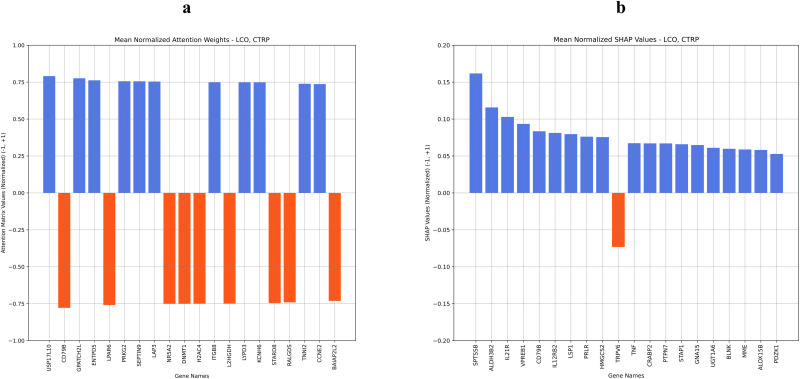
The plots present the sorted mean attention weights (a) and the SHAP values (b), representing gene importance across all breast cancer cell lines in the CTRP datasets under the LCO setup. Higher values indicate a greater contribution to the model’s decision-making process.

We used SHAP values and attention weights as gene importance scores for a representative subset of BC cell lines. In Fig 8 and S26 Fig in S1 File, the top 20 genes (ranked by importance), along with their importance scores and directionality of effect, are presented for each explainability method and dataset.

According to the attention-based model in the CTRP dataset, the highest-ranked gene was *USP17L10* (Ubiquitin Specific Peptidase 17-Like Family Member 10). It encodes a predicted cysteine-type deubiquitinase enzyme. Such deubiquitinating enzymes regulate cell proliferation, cell-cycle progression, apoptosis, cell migration, and antiviral responses [55]. By contrast, the RankingSHAP analysis identified *SPTSSB* (Serine Palmitoyltransferase Small Subunit B) as a crucial gene in sphingolipid metabolism. Studies have shown that nearly all BC subtypes exhibit distinct and significant alterations in sphingolipid metabolism. Changes in sphingolipid metabolites, such as ceramides, sphingosine, and sphingomyelin, as well as modifications in their biosynthetic and catabolic enzymatic pathways, have emerged as key molecular mechanisms that influence BC cell growth, response to therapy, and resistance to treatment. These alterations may also hold diagnostic and prognostic significance [56].

In the PRISM dataset, the gene *CLEC7A* (C-type Lectin Domain Containing 7A) was most important according to the attention-based model, whereas *RARRES1* (Retinoic Acid Receptor Responder 1) was top-ranked by RankingSHAP. *CLEC7A* is implicated in glioma progression and is considered an independent prognostic factor in that context [57]. *RARRES1* is another important gene, as its expression levels vary significantly among different BC subtypes. According to a recent study, *RARRES1* serves as a specific prognostic predictor for Triple-Negative Breast Cancer (TNBC) [56].

**Fig 9:**
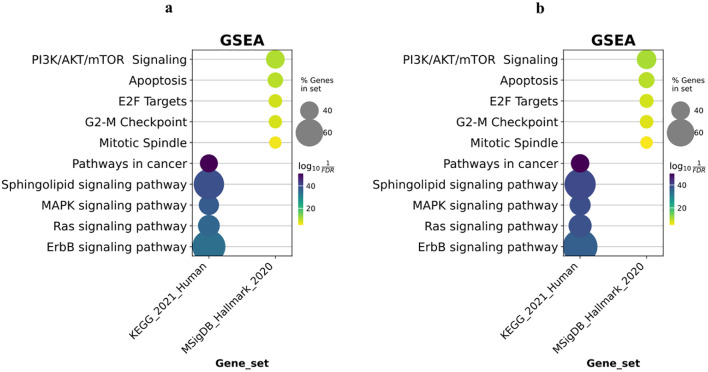
The figures illustrate the GSEA analysis of the model’s attention matrix values (a) and SHAP values (b) for cell lines in the CTRP dataset under the LCO setup, revealing key biological pathways that may influence the model’s predictions.

To further interpret the significance of these gene features, we performed gene set enrichment analysis (GSEA) to identify biological pathways associated with the genes prioritized by each explainability method. Genes with nonzero importance scores (averaged across all BC cell lines) were pooled for each technique and tested for enrichment against the KEGG 2021 Human pathways and the MSigDB Hallmark 2020 gene sets [58,59]. The complete GSEA results are provided in the Supporting Information, and Fig 9 and S27 Figs in S1 File highlight the most significantly enriched pathways for each dataset and interpretability approach.

Overall, both interpretability methods identified major cancer-related pathways. These included Apoptosis, general Pathways in Cancer, the G2/M Checkpoint, Chemical Carcinogenesis, and *PI3K/AKT/mTOR* Signaling. Inflammatory pathways that drive tumor progression were also enriched, such as Cytokine-Cytokine Receptor Interaction, *TNF-α* Signaling, and the broad Inflammatory Response. Additionally, pathways more specific to BC biology, namely Calcium Signaling and Cholesterol Homeostasis, were detected by the models. Calcium signaling is known to be dysregulated in aggressive BC subtypes like *TNBC* and *HER2*-positive disease; alterations in calcium channels, pumps, and calcium-binding proteins can promote cancer cell survival, drug resistance, and metastasis [60]. Cholesterol metabolism also plays a critical role in cancer progression: for example, the low-density lipoprotein receptor (*LDLR*) regulates cellular cholesterol homeostasis and is an independent prognostic factor in BC [61].

When stratifying the analysis by Estrogen Receptor (ER) status, RankingSHAP captured more subtype-specific pathways than the attention-based model. In ER-positive cell lines, RankingSHAP highly ranked estrogen-related pathways (e.g., Estrogen Response Early, Estrogen Response Late, and Estrogen Signaling), whereas in ER-negative cell lines it highlighted more general oncogenic and inflammatory pathways (see Table 4). The attention-based approach, in contrast, did not show a clear distinction between ER-positive and ER-negative subgroups, failing to emphasize the estrogen-signaling pathways in ER-positive lines (see S5 Table in S1 File).

**Table 4:** GSEA analysis of ER+ and ER- breast cancer cell lines using RankingSHAP to identify key biological pathways associated with gene importance.

Gene_set	Term	P-value	Adjusted P-value	Cell Line ER
MSigDB_Hallmark_2020	Estrogen Response Early	8.81E-09	3.44E-07	Positive
MSigDB_Hallmark_2020	Estrogen Response Late	2.15E-04	4.19E-03	Positive
MSigDB_Hallmark_2020	Peroxisome	9.41E-04	1.22E-02	Positive
MSigDB_Hallmark_2020	Inflammatory Response	3.17E-03	2.48E-02	Positive
MSigDB_Hallmark_2020	Xenobiotic Metabolism	3.17E-03	2.48E-02	Positive
KEGG_2021_Human	PPAR signaling pathway	1.44E-07	3.71E-05	Positive
KEGG_2021_Human	Cytokine-cytokine receptor interaction	5.07E-07	6.54E-05	Positive
KEGG_2021_Human	Estrogen signaling pathway	1.02E-06	8.74E-05	Positive
KEGG_2021_Human	Calcium signaling pathway	3.41E-06	1.80E-04	Positive
KEGG_2021_Human	Oxytocin signaling pathway	3.50E-06	1.80E-04	Positive
MSigDB_Hallmark_2020	Inflammatory Response	7.45E-10	3.20E-08	Negative
MSigDB_Hallmark_2020	TNF-alpha Signaling via NF-kB	2.52E-08	5.41E-07	Negative
MSigDB_Hallmark_2020	KRAS Signaling Up	1.34E-07	1.92E-06	Negative
MSigDB_Hallmark_2020	Epithelial Mesenchymal Transition	6.74E-07	7.25E-06	Negative
MSigDB_Hallmark_2020	Myogenesis	5.76E-05	4.95E-04	Negative
KEGG_2021_Human	Neuroactive ligand-receptor interaction	1.63E-14	4.47E-12	Negative
KEGG_2021_Human	Cytokine-cytokine receptor interaction	6.50E-11	8.90E-09	Negative
KEGG_2021_Human	PI3K-Akt signaling pathway	2.52E-07	2.00E-05	Negative
KEGG_2021_Human	Steroid hormone biosynthesis	3.56E-07	2.00E-05	Negative
KEGG_2021_Human	Rheumatoid arthritis	3.64E-07	2.00E-05	Negative

Finally, we compared the important genes identified by each method with genomic mutation data for these cell lines. For each cell line, we collected genes whose importance score was at least one standard deviation above the mean and checked their overlap with the set of non-silent mutated genes in that cell line. The attention-based model’s high-importance genes overlapped with known mutated genes in approximately 36% of cases, whereas the RankingSHAP method’s top genes showed only about 13% overlap.

#### 3.11.2 Molecules explainability.

To better understand how the models make predictions on molecular structures, RankingSHAP was used to identify which molecular substructures contribute most to the model’s decisions. The feature output is hashed since Morgan and AtomPair fingerprints are employed as molecular representations. We used the bit-info dictionary to trace each feature vector’s structural origin. However, a key limitation of these fingerprint-based representations is hash collision, meaning that different molecular substructures may be mapped to the same hashed feature. This overlap reduces the accuracy of atom- and substructure-level attribution.

**Fig 10:**
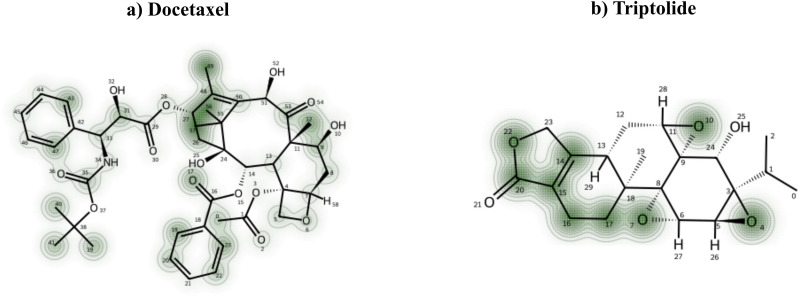
These two figures present the molecule structures of Docetaxel (a) and Triptolide (b) with contours that highlight the model’s points of interest based on SHAP values. The larger the highlighted area, the greater the impact on the model’s predictions.

To partially mitigate this issue, we apply a filtering threshold of |SHAP value| ≥ mean + 4 × standard deviation, ensuring that only the most significant molecular features are retained. The appearance frequency of each atom within these high-importance features is then counted using the bit-info dictionary, followed by normalization. The resulting importance scores are mapped onto the molecular structure, as illustrated in Fig 10.

In this section, we evaluate two top-ranked drugs based on the model’s explainability outputs and their Structure-Activity Relationship (SAR).

#### 3.11.3 Docetaxel: A key drug in BC chemotherapy.

Docetaxel, a taxane-class chemotherapeutic agent, is a standard component of many BC treatment regimens. The model consistently ranked it among the top five most effective drugs across multiple BC cell lines.

SAR studies have established that the side chain bonded to C-27 plays a crucial role in taxane bioactivity. Additionally, the A-ring structure is conserved across taxanes, including Paclitaxel, reinforcing its importance in drug function. Furthermore, the N-acetyl group (atoms 34, 35, 36, and 37) is critical for activity, along with the acyloxy group that binds to the main ring. All of these functionally vital substructures were correctly identified by RankingSHAP.

Conversely, less functionally relevant sections, such as hydroxyl groups OH-25 and OH-52, were assigned low importance, aligning with prior biological knowledge [62].

#### 3.11.4 Triptolide: A potential emerging therapy.

Triptolide emerged as one of the top-ranked drugs predicted by the best-performing models across multiple BC cell lines. Although not yet FDA-approved for BC treatment, an ongoing Phase I clinical trial (ClinicalTrials.gov) is evaluating its safety and efficacy in BC under the brand name Minnelide™.

SAR analysis indicates that three epoxide groups (atoms 10, 4, and 7) are essential for Triptolide’s biological activity, while the unsaturated lactone ring contributes to drug effectiveness. RankingSHAP correctly highlighted these critical substructures, as shown in Fig 10a [63].

In the following section, we discuss the results obtained from the experiments and propose several future research directions for interested researchers.

## 4 Discussion

This study highlights the critical role of both ranking loss functions and molecule representations in driving model performance. In the CTRP dataset, we observed that using LambdaLoss led to the best overall ranking, whereas in PRISM, LambdaRank emerged as the top performer. This shows that the methodological choice to evaluate six loss functions alongside five distinct molecule representations was essential for uncovering performance nuances. The comprehensive approach of using Friedman ranking and averaging across multiple metrics allowed us to discern subtle differences that would have been obscured by the wide ranges of evaluations, thereby hinting at selecting the most effective setups.

Based on the metric-wise results, the LambdaLoss and LambdaRank outperform the other loss functions. This indicates that incorporating the importance of misordering alongside the quality of the ranking list by focusing on a standard framework such as NDCG is far more effective than solely assessing model predictions based on the order of items’ probability of being effective (as with ListOne or ListAll). Additionally, the performance of these loss functions depends heavily on several hyperparameters. For instance, while the Pair-PushC loss function accounts for a trade-off between quality and item relevancy, finding the optimal value for α remains challenging for each task. Similarly, NeuralNDCG relies significantly on its temperature parameter to smooth its learning curve and achieve optimal performance.

The sensitivity analysis indicates that while precise hyperparameter tuning is necessary to achieve peak performance, the underlying architecture of the list-wise ranking framework remains remarkably robust across varying configurations. The model’s success is primarily driven by the inherent strengths of list-wise objective functions, which prioritize the relative hierarchy of drug responses, rather than an over-reliance on narrow parameter settings. This architectural stability is a critical feature for generalizability, mitigating the risk of overfitting to dataset-specific noise and ensuring reliable performance when transitioning to unseen or noisier biological datasets where exact parameter alignment may be elusive. Ultimately, this resilience suggests the framework captures fundamental biological patterns of drug sensitivity, making it a dependable candidate for other computational biology tasks and adaptation to new pharmacological domains without requiring exhaustive computational overhead for re-optimization.

Moreover, the metric-wise analysis confirms the earlier discussion. Across various values of K, LambdaLoss consistently outperforms its peers, with LambdaRank and ListOne following closely. However, it is noteworthy that while early ranking metrics favored these loss functions, certain configurations, such as NeuralNDCG, exhibited weaknesses, particularly in the LRO scenario with the CTRP dataset. These results justify the decision to incorporate multiple ranking loss functions in the study, as they reveal the trade-offs between overall item relevancy (CI) and early ranking quality (AP, AH, and NDCG). Overall, the analysis confirms that optimizing the loss function is crucial for achieving specific performance goals, depending on the dataset and the evaluation scenario.

For molecule representations, the most obvious finding is that fingerprints capture atomic features better than descriptors, as the results of the ablation study. This suggests that the structural features of a molecule are more critical than its physicochemical properties for drug response prediction tasks. However, combining these two representations can help the model better understand drug characteristics. Specifically, the AtomPair and Morgan fingerprints, with or without descriptors, consistently achieve top performance in nearly every setting. In contrast to LayeredRDKit, these two fingerprints capture comprehensive substructural features and local atomic properties rather than treating atoms and bonds as separate layers. In summary, the overall structural features of a molecule, combined with its physicochemical properties, provide a more complete representation.

In contrast to the stark differences observed with loss functions, the impact of molecule representations was more subtle. The findings indicate that while performance trends remain consistent across different Ks, the choice of representation has a less dramatic influence than that of the loss function. For instance, the AtomPair and Morgan fingerprints, along with other fingerprint types, showed only marginal performance differences across datasets and evaluation setups. This observation suggests that while selecting an appropriate molecular representation or descriptor is important, fine-tuning the loss function may yield greater benefits.

Furthermore, our explicit evaluation under controlled imbalance conditions demonstrates that ranking loss functions maintain stable performance across a broad spectrum of effective-to-ineffective drug ratios. Despite the inherent skewness of drug response datasets, NDCG scores varied only within a narrow range as the imbalance parameter α shifted from strongly ineffective-dominated to strongly effective-dominated sets. This robustness suggests that listwise and ranking based objectives are inherently well-suited for imbalanced drug sensitivity prediction tasks. Importantly, the stability across imbalance levels reinforces the practical applicability of these methods in real world scenarios, where the proportion of responsive drugs per cell line is typically small and highly variable.

Moreover, the qualitative evaluation of drug prioritization further supports these findings. The best-performing models, particularly those optimized with LambdaLoss, demonstrated clear disease-specific ranking behavior, consistently prioritizing clinically relevant drugs at higher positions for their corresponding cancer types. In contrast, the worst-performing models produced more dispersed and less biologically coherent rankings, indicating weaker discrimination between NSCLC and melanoma. These observations reinforce the metric-based results by showing that improvements in ranking metrics are not merely numerical gains, but translate into more meaningful and clinically aligned drug prioritization.

Nevertheless, this validation experiment represents a simplified and partially idealized scenario. The analysis maps clinically established therapies onto preclinical cell line models, which do not fully capture the complex biological, pharmacodynamic, and microenvironmental factors influencing drug efficacy in patients. Consequently, some inconsistencies observed even in the best-performing models may reflect intrinsic limitations of the experimental framework rather than shortcomings of the ranking approach itself.

Evaluating model generalizability across datasets provided valuable insights into the robustness of the selected approaches. Models trained on the CTRP dataset generally delivered higher early ranking quality metrics (AP, AH, NDCG), whereas those trained on PRISM achieved superior overall item relevancy, as measured by the concordance index. This difference can be attributed to the fact that CTRP contains more cell lines than PRISM, while PRISM includes a larger number of drugs. This divergence underscores a trade-off between item relevancy and overall ranking consistency, which is critical when deploying models in real-world scenarios. Furthermore, according to the definitions of the evaluation scenarios, the goal of the LCO setup is to keep a subset of cell lines unseen by the model, resulting in better generalizability compared to the LRO scenario. Overall, the generalizability study validates the importance of not only selecting the right loss function and molecular representation but also aligning the appropriate training dataset and scenario with the intended application domain to optimize performance on unseen data.

The interpretability results indicate that the ranking of drugs is largely influenced by biologically and chemically relevant features. Both the post-hoc approach of RankingSHAP (including the proposed modified version for query explainability) and the ad-hoc attention-based interpretability method are effective tools for evaluating model performance.

While RankingSHAP, and particularly its modified version, requires more computational resources than the attention-based model, especially in high-dimensional drug response tasks, its key advantage is that it can be applied to a pre-trained model without requiring retraining. This is particularly useful in cases where adding attention mechanisms negatively impacts model performance. In some cases, the attention mechanism fails to capture the key molecular patterns necessary for accurate ranking, likely due to sparsity in molecular feature vectors and the inherently fragmented nature of drug representations.

Both post-hoc and ad-hoc explainability methods successfully identified key cancer-related genes in BC cell lines. Since this study focused specifically on BC, many BC-specific genes were ranked highly in terms of importance. The attention-based approach demonstrated a stronger correlation with known mutated genes in cell lines, whereas RankingSHAP was more effective in capturing pathways that are specific to each ER subtype of BC. These findings suggest that the two interpretability approaches offer complementary insights into the model’s predictions. Using both methods together thus provides a more robust and nuanced understanding of the biological features driving drug response in BC cell lines.

The three-fold difference in mutation overlap between the two explainability methods (attention mechanism: 36% vs RankingSHAP:13%) suggests that the attention mechanism, as an intrinsic component trained end-to-end, is highly sensitive to high-variance features such as genomic alterations. In contrast, RankingSHAP is a post hoc, perturbation-based approach. Owing to the high dimensionality of the data and associated computational costs, a limited background sample size (four for genes and two for drugs) was used to ensure memory efficiency. This constraint likely reduces its ability to capture rare, sample-specific outlier mutations compared with the globally optimized attention mechanism. However, RankingSHAP identifies a broader set of genes that influence drug response through coordinated pathway-level activity rather than isolated mutations. This observation is supported by our GSEA, in which RankingSHAP consistently highlights biologically meaningful pathways. Thus, while the attention mechanism excels at pinpointing specific genetic drivers, RankingSHAP provides a more stable and systemic view of cell-line behavior.

Applying explainability techniques to drug molecules poses inherent challenges. Hash collisions in fingerprint-based molecular representations reduce the precision of feature attribution, as multiple atoms or substructures may be mapped to the same hashed feature. Moreover, high-dimensional sparse fingerprint vectors do not fully preserve spatial arrangement or stereochemical context, further limiting interpretability at fine resolution. In addition, the SARs of many compounds remain incompletely characterized, restricting the extent of independent biological validation and limiting definitive conclusions about which substructures drive efficacy, selectivity, or other pharmacological properties. Consequently, drug-side explanations are more appropriately interpreted at the level of influential substructures rather than definitive atom-level causality. Despite these constraints, model interpretability still provides valuable insights into how predictions are formed and which atomic features influence performance. Notably, in many cases, the highlighted substructures align well with literature-reported SAR evidence, further supporting the validity of the explainability approach. Future improvements in molecular representations, particularly methods that better preserve structural and stereochemical context, are likely to enhance the fidelity and granularity of drug-side interpretability in ranking models.

Future research should explore the integration of Graph Neural Networks (GNNs), particularly Message Passing Neural Networks (MPNNs), which show promise in capturing rich molecular structural and physicochemical properties for drug ranking tasks. While preliminary results indicated strong compatibility between MPNNs and LambdaRank-based loss functions, computational limitations prevented a full exploration. Efficient training strategies and scalable architectures will be crucial for leveraging GNNs in this context. Furthermore, expanding drug ranking frameworks to include combination therapies is a key direction, as most clinical regimens involve multi-agent treatments. Incorporating datasets with drug combination dose-response curves could enhance predictive accuracy, though doing so requires addressing challenges like modeling additive, synergistic, and antagonistic effects. Additionally, as more biologically relevant cancer models such as organoids and patient-derived xenografts (PDX) emerge, future models must support multi-source learning to bridge the gap between in vitro, ex vivo, and clinical scenarios, despite the scarcity of high-quality data in these systems. Finally, interpretability remains an underexplored area in LeToR models. Although tools like RankingSHAP and our proposed attention-based method offer meaningful insights, there is a pressing need for more robust, ranking-specific explainability techniques. Such methods could advance biological understanding by uncovering key genes, pathways, and molecular substructures involved in drug response, ultimately aiding in precision oncology and drug discovery.

## Supporting information

S1 File Shedding light on neural learning to rank models for anticancer drug prioritization. This file is provided as supplementary material and includes all supporting information cited throughout the manuscript.
(DOCX)

S2 Table Results of all experiments. This file presents the complete results of all experiments conducted in this study.
(CSV)
