# Supplementary material for: Shedding light on neural learning to rank models for anticancer drug prioritization
Source: PLoS One. 2026 Aug 13;21(8):e0345854. doi: 10.1371/journal.pone.0345854 (PMC13472410; doi:10.1371/journal.pone.0345854)
Supplement: Supplementary file 1 [file pone.0345854.s001.docx]

**Shedding light on neural learning to rank models for anticancer drug prioritization**

Faraz Sarmeili^1,2^, Benyamin Ghahremani-Nezhad^3^, Mohammad Khalilpour^3^, Karim Abbasi^4^, Rassoul Dinarvand^1,5 *^, Hamid R. Rabiee^2,*^

^1^Department of Pharmaceutics, Faculty of Pharmacy, Tehran University of Medical Sciences, Tehran, Iran, [sarmeili.f@gmail.com](mailto:sarmeili.f@gmail.com), [dinarvand@tums.ac.ir](mailto:dinarvand@tums.ac.ir).

^2^Department of Computer Engineering, Sharif University of Technology, Tehran, Iran, [sarmeili.f@gmail.com](mailto:sarmeili.f@gmail.com), [rabiee@sharif.edu](mailto:rabiee@sharif.edu).

^3^Department of Computer Engineering, Amirkabir University of Technology, Tehran, Iran, [benjaminghahremani@gmail.com](mailto:benjaminghahremani@gmail.com?to=%22Benyamin%20Ghahremani%20Nezhad%22%20%3Cbenjaminghahremani%40gmail.com%3E), [kian.khalilpour@gmail.com](mailto:kian.khalilpour@gmail.com).

^4^Mosaheb Institute for Mathematical Research, Kharazmi University, Tehran, Iran, [karim.abbasi@ut.ac.ir](mailto:karim.abbasi@ut.ac.ir).

^5^ Nanotechnology Research Centre, Faculty of Pharmacy, Tehran University of Medical Sciences, Tehran, Iran, [dinarvand@tums.ac.ir](mailto:dinarvand@tums.ac.ir).

* Corresponding Author

Email: [dinarvand@tums.ac.ir](mailto:dinarvand@tums.ac.ir)

Email: [rabiee@sharif.edu](mailto:rabiee@sharif.edu)

# **S1 Supplementary Materials**

## **S1.1 Performance Metrics**

The performance of each model is evaluated using ranking metrics commonly adopted in the information retrieval community. Although these metrics are sometimes correlated, each captures different aspects of ranking quality. For example, some metrics evaluate the overall ordering of items, whereas others focus primarily on the relevance of the top-ranked items. For each model, the average value of each metric is reported over all queries (in our case, queries correspond to different cell lines). Below are descriptions of the metrics used:

### **S1.1.1 Normalized Discounted Cumulative Gain at K (NDCG@K)**

Normalized Discounted Cumulative Gain (NDCG) is a widely used metric for evaluating the quality of ranked results. It quantifies the gain a user receives from viewing documents in a ranked list, while applying a logarithmic discount factor to account for the fact that users are more likely to examine items at the top of the list.

At its core, NDCG relies on the computation of Discounted Cumulative Gain (DCG), typically defined as:

$$DCG@K=\sum_{i=1}^{k} \frac{{rel}_{i}}{\log_{2} (i+1)}$$

**(S1)**

where ${rel}_{i}$ represents the graded relevance score of the document at position i, and k is the ranking cutoff. This formulation ensures that documents ranked lower contribute less to the overall score.

To enable fair comparisons across different queries or ranking scenarios, DCG is normalized by the Ideal Discounted Cumulative Gain (IDCG), which is computed using the optimal ordering of documents (i.e., sorted by decreasing relevance). The normalized metric is given by:

$$NDCG@K=\frac{DCG@K}{IDCG@K}$$

**(S2)**

By construction, NDCG scores range from 0 to 1, with a score of 1 indicating a perfectly ordered list where the most relevant documents appear at the top [1].

### **S1.1.2 Average Precision (AP@K)**

Average Precision (AP) is a common metric for assessing the effectiveness of ranking systems by considering both the order and the relevance of retrieved documents. It computes the average precision values at each rank position where a relevant document is retrieved, thereby rewarding systems that retrieve relevant items and rank them higher in the list.

Mathematically, AP@K is expressed as:

$$AP@K=\frac{1}{N}\sum_{i=1}^{K} P_{i}* {rel}_{i}$$

**(S3)**

where $P_{i}$ is the precision at position i, ${rel}_{i}$ is a binary indicator that is 1 if the document at position *i* is relevant (and 0 otherwise), and *N* is the total number of relevant items within the top *K* results. The AP score ranges from 0 to 1, with 1 indicating that all relevant documents are retrieved at the highest ranks.

### **S1.1.3 Average Hit (AH@K)**

Average Hit (AH) is a more straightforward metric that focuses solely on the number of relevant items retrieved in the top *K* results, without considering the order in which they appear. It simply counts the number of relevant items in the top *K* list. Therefore, AH@K can take any value from 0 up to *K*.

### **S1.1.4 Concordance Index (CI)**

The Concordance Index (CI) measures a model’s ability to correctly order pairs of instances based on their predicted scores relative to the observed outcomes, thereby reflecting the discrimination power of the model. It is calculated as follows:

$$CI=\frac{\sum CP}{\sum EP}$$

**(S4)**

where *CP* represents the number of concordant pairs (i.e., pairs of instances for which the ordering of predicted scores agrees with the ordering of observed outcomes), and *EP* is the total number of evaluable pairs. CI values range between 0 and 1, with a value of 0.5 indicating performance comparable to random prediction [2].

## **S1.2 Molecule Representations**

There are various ways to represent drugs computationally in drug response prediction tasks. These representations assign each drug an embedding or a feature vector that captures specific molecular characteristics. Some methods focus on chemical properties, others emphasize structural features, and some combine using different computational techniques. Broadly, these methods can be divided into four categories:

- Fingerprints primarily encode structural features by counting a molecule's distinct substructures or other structural elements.
- Descriptors: These capture the overall physicochemical properties of entire molecules.
- Sequence Encoding: These methods use the SMILES representation of molecules and apply natural language processing (NLP) techniques to learn embeddings by uncovering correlations between SMILES strings and drug response.
- Molecular Graphs: This advanced approach leverages graph neural network (GNN) architectures to embed the chemical and structural features of atoms, bonds, and the whole molecule.

This article focuses on fingerprints and reports experiments using atom-pair fingerprints, RDKit-layered fingerprints, and Morgan fingerprints. As described above, fingerprints capture detailed structural features, whereas descriptors provide a broader view of a molecule’s chemical properties [3, 4]. Based on this complementary information, we propose combining both representation methods by concatenating fingerprints with descriptors. In the following sections, we describe each molecular representation method in detail.

### **S1.2.1 Morgan fingerprint**

Morgan fingerprints provide a robust and computationally efficient way to represent and analyze chemical structures. They have become essential tools in predicting drug response and aiding pharmaceutical development. Their speed and efficiency make them well-suited for high-throughput screening in pharmaceutical research, and they are increasingly used in machine learning tasks such as property prediction and drug repurposing [5, 6].

In the Morgan method, a molecule is treated as a graph in which atoms are nodes and bonds are edges. The process begins by assigning an initial identifier (or invariant) to each atom based on properties such as atomic number, hybridization state, valence, and other chemical attributes. Next, each atom’s identifier is iteratively updated by combining it with the identifiers of its neighboring atoms. The algorithm typically uses a hash function in each iteration to merge an atom’s current identifier with its directly connected neighbors. The number of iterations, often called the “depth” or “radius”, is fixed beforehand. After completing the iterative updates, the resulting identifiers (which now capture circular substructures around each atom) are sorted to produce a canonical representation of the molecule. Finally, these sorted identifiers are hashed into a fixed-size feature vector suitable for machine learning applications [7, 8].

### **S1.2.2 AtomPair fingerprint**

The atom‐pair fingerprint is critical in Quantitative Structure-Activity Relationship (QSAR) studies. Its primary advantage is that it considers every pair of non‐hydrogen atoms within a molecule, providing a more comprehensive topological representation of the structure. Although atom‐pair fingerprints are widely used in chemoinformatics and computational biology, they are less frequently applied in drug response prediction than circular fingerprints like Morgan, which are more efficient in capturing local substructures with lower dimensionality.

Atom‐pair fingerprints encode a molecule’s structure by accounting for the atom types and the shortest‐path distances between them. In practice, the method involves enumerating every unique pair of atoms in the molecule and recording the number of bonds (i.e., the topological distance) that separate them. This approach captures both local interactions (involving adjacent or nearby atoms) and long‐range interactions (between atoms that are farther apart), making it particularly suitable for representing structurally diverse datasets. Once all relevant atom pairs and their distances are determined, the information is hashed into a fixed‐size feature vector that represents the molecule [9].

### **S1.2.3 RDKit layered fingerprint**

Both Morgan and atom-pair fingerprints and the descriptor described in the following section are implemented within the RDKit package for Python. RDKit is a powerful chemoinformatics toolkit consolidating many computational chemistry tools into one platform. Consequently, we used the built-in RDKit layered fingerprint in our experiments. This fingerprint is designed to capture substructural features using a layered-based approach. Rather than uniformly hashing all molecular substructures, the layered fingerprint first decomposes a molecule into its constituent subgraphs (typically bond paths within a specified length). Then it hashes each subgraph using a scheme that selectively incorporates multiple layers of chemical information (e.g., connectivity, bond order, atom types, ring membership, ring sizes, and aromaticity) according to user-defined layer flags. The resulting hash values are folded into a fixed-length bit vector (typically 2048 bits), yielding a compact, tunable descriptor for machine learning and other computational tasks [10, 11].

### **S1.2.4 Descriptor**

We utilized the built-in RDKit molecule descriptor calculator for the molecule descriptor, which aggregates the calculation of multiple molecular descriptors into a single workflow. In practice, it iterates over a predefined list of descriptor functions, such as those for molecular weight, topological polar surface area (TPSA), LogP, and the number of rotatable bonds. It applies each function to a given molecule. The results are then returned as a numerical feature vector that encapsulates the molecule's various physicochemical and structural properties. This automated aggregation streamlines the process of converting chemical structures into numerical data, which is essential for QSAR analyses and machine learning applications in cheminformatics [10].

## **S1.3 Attention Mechanism**

The attention mechanism was first introduced by [12] for the transformer model. The idea allows the model to weigh the influence of all other positions in the input sequence when encoding a given token, enabling the capture of long-range dependencies without the sequential bottlenecks of recurrent models. On the other hand, the self-attention block helps to improve the model's performance by finding intra-relationships of the features. Briefly, a self-attention block can be formulated as follows:

$$\text{Attention}\left( Q,K,V \right)=\text{softmax}\left( \frac{QK^{\top}}{\sqrt{d_{k}}} \right) V$$

**(S5)**

Where:

- Query (Q): A matrix representing the “queries,” which capture the current context or the element you want to focus on.
- Key (K): A matrix representing “keys,” which help match queries to the relevant information in the sequence.
- Value (V): A matrix representing “values,” which hold the actual information to be used or returned after matching queries and keys.
- $d_{k}$ is the dimensionality of the keys

In summary, the mechanism involves first projecting the input (e.g., word embeddings) into query, key, and value representations and then computing how similar each query is to each key by taking their dot product (scaled by the square root of the key dimensionality to stabilize training). A SoftMax function turns these scores into attention weights, which are used to weight the corresponding values, yielding a context-aware output.

## **S1.4 Gene Selection Method**

To enable model explainability, we first selected a subset of approximately 19,000 genes to interpret the outputs of our explainability methods efficiently. One widely used approach in the literature is network propagation on a protein-protein interaction (PPI) network, using the target protein(s) of drugs from the drug-response dataset. This method is well established in various areas of bioinformatics [13-15], and has been applied in several important drug response prediction studies [16-19].

The core concept of this method is to construct a PPI network using the STRING database, where each node represents a protein and each edge represents a relationship between two proteins. These edges are weighted according to the reliability of the interaction as derived from the literature. The combined interaction score is provided directly by the STRING database and is subsequently used in our network analysis [20].

Next, the target proteins corresponding to the drugs in our dataset are identified within the PPI network. In this network, the target proteins are assigned an initial weight of 1, while all other proteins receive a minimal weight (10e-5) in the initial weight vector $W_{0}$.

The weight vector is then iteratively updated using the following equation:

$W_{t+1}=\alpha W_{t}A^{'}+\left( 1- \alpha\right)W_{0}$	where	$A^{'}=D^{-\frac{1}{2}}AD^{-\frac{1}{2}}$

Here, *A* is the adjacency matrix of the network, *D* is the degree matrix, and $A^{'}$ is the normalized adjacency matrix. We utilized the weighted edges in A to better identify the most important proteins. The parameter $\alpha$ ($\mathrm{with} 0 \leq\alpha\leq1)$ known as the diffusion parameter, controls the extent to which the initial information diffuses throughout the network. Based on previous studies, a value of $\alpha=0.7$ has been shown to select the most relevant proteins effectively. The iterative process continues until convergence, defined as ${||W}_{t+1}-W_{t}||\leq1e-6$.

This process simulates a random walk originating from the initial drug target nodes. The resulting weight vector W is sorted for each drug, and the top 20 genes are selected. These genes are then used for further interpretation and as features in gene expression analysis.

## **S1.5 Loss Function**

Below are the three main parts of the PairPushC loss function and their assembly breakdown:

- Drug Classification Loss

This loss term is introduced to improve the separation between effective and ineffective drugs by training an auxiliary classifier S. For each drug d in cell line c, the classifier outputs the probability $\hat{y}_{d}$ that the drug is effective, the loss is defined as a binary cross‐entropy considering all drugs in every cell line:

$$\mathcal{B}_{\mathcal{S}} = \sum_{c\in\mathcal{C}} \left[ \sum_{d\in\mathcal{D}_{\mathcal{c}^{+}}} \log\left( \hat{y}_{d} \right) + \sum_{d\in\mathcal{D}_{\mathcal{c}^{-}}} \log\left( 1-\hat{y}_{d} \right) \right]$$

**(S6)**

$\mathcal{C}$ is the set of all cell lines.

$\mathcal{D}_{\mathcal{c}^{+}}$ is the set of effective drugs for cell line c.

$\mathcal{D}_{\mathcal{c}^{-}}$ is the set of ineffective drugs for cell line c.

$\hat{y}_{d}$ is the predicted probability that drug d is effective in cell line c.

- “push” Mechanism in pLETORg

In pLETORg, the idea is to “push” the sensitive drugs to the top of the ranking list in each cell line. The method can be defined and formalized as follows:

$$P_{f}^{\uparrow} = \sum_{p=1}^{m} \frac{1}{n_{p}^{+} n_{p}^{-}} \sum_{d_{i}^{-}\in C_{p}} h_{f}\left( d_{i}^{-},C_{p} \right)$$

**(S7)**

In this equation, for each cell line $C_{p}$ (with m cell lines), the term $h_{f}\left( d_{i}^{-},C_{p} \right)$ counts the number of sensitive drugs that are ranked below an insensitive drug $d_{i}^{-}$. The sum is normalized by the total number of sensitive-insensitive pairs $n_{p}^{+} n_{p}^{-}$ for that cell line. Minimizing $P_{f}^{\uparrow}$ encourages the model to “push” sensitive drugs above insensitive ones.

- Objective for Ranking among Sensitive Drugs

In addition to pushing sensitive drugs above insensitive ones, the mechanism also refines the ranking among the sensitive drugs themselves. This ranking approach helps ensure that the most promising drugs are selected and accurately prioritized relative to one another. Here's the formula for this method:

$$O_{f}^{+} = \sum_{p=1}^{m} \left[ 1-CI\left( \{d_{i}^{+}\},C_{p},f \right) \right] = \sum_{p=1}^{m} \frac{1}{\left| \{d_{i}^{+}\succ_{C_{p}}d_{j}^{+}\} \right|} \sum_{d_{i}^{+}\succ_{C_{p}}d_{j}^{+}} I\left( d_{i}^{+}\prec_{f}d_{j}^{+} \right)$$

**(S8)**

This term measures the ranking error among only the sensitive drugs. The first expression computes the sum over cell lines of $1 - CI$ (with CI being the concordance index among sensitive drugs). Equivalently, it can be written as a normalized sum over all misordered sensitive drug pairs, where $\left( d_{i}^{+}\prec_{f}d_{j}^{+} \right)$ indicates that a sensitive drug $d_{i}^{+}$ is incorrectly ranked below another sensitive drug $d_{j}^{+}$.

By jointly optimizing these three components, Pair-PushC balances the goals of (a) enhancing discrimination between effective and ineffective drugs, (b) pushing effective drugs above ineffective ones, and (c) refining the order among the effective drugs themselves. The hyperparameter $\alpha$ is typically tuned (e.g., via grid search) to find the best trade-off between these objectives.

# **S2 Supplementary Experiments**

**Table S1. Description of Datasets**

| Dataset | # Cell Lines | # Drugs | #AUCs | %missing drugs/cell line |
| --- | --- | --- | --- | --- |
| CTRP | 809 | 545 | 357544 | 18.9 |
| PRISM | 476 | 1446 | 617882 | 10.2 |

**Fig S1. Hyperparameter Sensitivity Analysis for LambdaLoss. The heatmaps display the impact of** $\boldsymbol{\mu}$ **and σ on (a) AP@10-20 and (b) NDCG@10-20**

**Fig S2. Hyperparameter Sensitivity Analysis for LambdaRank. The heatmaps display the impact of** $\boldsymbol{\mu}$ **and σ on (a) AP@10-20 and (b) NDCG@10-20**

**Fig S3. Hyperparameter Sensitivity Analysis for PairPushC. The heatmaps display the impact of α and β on (a) AP@10-20 and (b) NDCG@10-20.**

**Fig S4. Hyperparameter Sensitivity Analysis for ListAll. The plots illustrate the effect of the m parameter on (a) AP@10-20 and (b) NDCG@10-20**

**Fig S5. Hyperparameter Sensitivity Analysis for ListOne. The plots illustrate the effect of the m parameter on (a) AP@10-20 and (b) NDCG@10-20**

**Fig S6. Hyperparameter Sensitivity Analysis for NeuralNDCG. The plots illustrate the effect of the temperature parameter on (a) AP@10-20 and (b) NDCG@10-20**

**Fig S7. The plots illustrate the average Friedman ranking of all performance metrics for the experiments in the CTRP (a) and PRISM (b) datasets for the LRO setup.**

**Fig S8. Statistical comparison of model rankings for small list sizes (K = 1, 3, 5) under the LCO setup using the CTRP dataset. (a) Friedman ranking of models, including the corresponding Friedman test chi-square statistic. (b) P-value matrix from the Nemenyi post-hoc test showing pairwise differences in model performance.**

**Fig S9. Statistical comparison of model rankings for medium list sizes (K = 10, 20) under the LCO setup using the CTRP dataset. (a) Friedman ranking of models, including the corresponding Friedman test chi-square statistic. (b) P-value matrix from the Nemenyi post-hoc test showing pairwise differences in model performance.**

**Fig S10. Statistical comparison of model rankings for large list sizes (K = 40, 60) under the LCO setup using the CTRP dataset. (a) Friedman ranking of models, including the corresponding Friedman test chi-square statistic. (b) P-value matrix from the Nemenyi post-hoc test showing pairwise differences in model performance.**

**Fig S11. Statistical comparison of model rankings for small list sizes (K = 1, 3, 5) under the LCO setup using the PRISM dataset. (a) Friedman ranking of models, including the corresponding Friedman test chi-square statistic. (b) P-value matrix from the Nemenyi post-hoc test showing pairwise differences in model performance.**

**Fig S12. Statistical comparison of model rankings for medium list sizes (K = 10, 20) under the LCO setup using the PRISM dataset. (a) Friedman ranking of models, including the corresponding Friedman test chi-square statistic. (b) P-value matrix from the Nemenyi post-hoc test showing pairwise differences in model performance.**

**Fig S13. Statistical comparison of model rankings for large list sizes (K = 40, 60) under the LCO setup using the PRISM dataset. (a) Friedman ranking of models, including the corresponding Friedman test chi-square statistic. (b) P-value matrix from the Nemenyi post-hoc test showing pairwise differences in model performance.**

**Fig S14. Statistical comparison of model rankings for small list sizes (K = 1, 3, 5) under the LRO setup using the CTRP dataset. (a) Friedman ranking of models, including the corresponding Friedman test chi-square statistic. (b) P-value matrix from the Nemenyi post-hoc test showing pairwise differences in model performance.**

**Fig S15. Statistical comparison of model rankings for medium list sizes (K = 10, 20) under the LRO setup using the CTRP dataset. (a) Friedman ranking of models, including the corresponding Friedman test chi-square statistic. (b) P-value matrix from the Nemenyi post-hoc test showing pairwise differences in model performance.**

**Fig S16.** **Statistical comparison of model rankings for large list sizes (K = 40, 60) under the LRO setup using the CTRP dataset. (a) Friedman ranking of models, including the corresponding Friedman test chi-square statistic. (b) P-value matrix from the Nemenyi post-hoc test showing pairwise differences in model performance.**

**Fig S17.** **Statistical comparison of model rankings for small list sizes (K = 1, 3, 5) under the LRO setup using the PRISM dataset. (a) Friedman ranking of models, including the corresponding Friedman test chi-square statistic. (b) P-value matrix from the Nemenyi post-hoc test showing pairwise differences in model performance.**

**Fig S18.** **Statistical comparison of model rankings for medium list sizes (K = 10, 20) under the LRO setup using the PRISM dataset. (a) Friedman ranking of models, including the corresponding Friedman test chi-square statistic. (b) P-value matrix from the Nemenyi post-hoc test showing pairwise differences in model performance.**

**Fig S19. Statistical comparison of model rankings for large list sizes (K = 40, 60) under the LRO setup using the PRISM dataset. (a) Friedman ranking of models, including the corresponding Friedman test chi-square statistic. (b) P-value matrix from the Nemenyi post-hoc test showing pairwise differences in model performance.**

**Fig S20. The spider-plots of evaluation metrics at different Ks for the CTRP (a) and PRISM (b) datasets in the LRO setup across all loss functions. The "SCL" prefix of AH is the short form of the word “Scaled”.**

**Fig S21. The spider plots of evaluation metrics at different Ks for the CTRP (a) and PRISM (b) datasets in the LRO setup across all molecule representations. The "SCL" prefix of AH is the short form of the word “Scaled”.**

**Fig S22 Robustness of ranking loss functions under varying degrees of data imbalance. The x-axis represents the imbalance parameter 𝛼, ranging from −1 (strong bias toward ineffective drugs) to 1 (strong bias toward effective drugs), while the y-axis shows the average NDCG computed over grouped cutoff levels of 𝐾. Each subfigure illustrates the performance of a different ranking loss function: (a) LambdaLoss, (b) LambdaRank, (c) NeuralNDCG, (d) ListAll, (e) PairPushC, and (f) ListOne.**

**Table S2. Models generalizability performance in the LRO setup. Highlighted values indicate the best performances. The first three rows are the models trained on CTRP and evaluated on the PRISM dataset. On the other hand, the second three rows are models trained on the PRISM dataset and evaluated on CTRP.**

| Loss Function | Molecule Representation | CI | AP  1-3-5 | AP  10-20 | AP  40-60 | AH  1-3-5 | AH  10-20 | AH  40-60 | NDCG  1-3-5 | NDCG  10-20 | NDCG  40-60 |
| --- | --- | --- | --- | --- | --- | --- | --- | --- | --- | --- | --- |
| LambdaLoss | Desc+AtomPair | 0.587 | **0.577** | **0.530** | **0.423** | **1.301** | **5.598** | 14.277 | **0.906** | 0.879 | 0.836 |
| LambdaLoss | Desc+Morgan | 0.608 | 0.520 | 0.498 | 0.413 | 1.181 | 5.514 | **14.675** | 0.904 | **0.892** | **0.877** |
| LambdaLoss | RDKitLayer | 0.586 | 0.524 | 0.481 | 0.373 | 1.120 | 4.678 | 13.316 | 0.900 | 0.860 | 0.825 |
| ListOne | Morgan | **0.630** | 0.181 | 0.267 | 0.272 | 0.405 | 3.285 | 11.731 | 0.665 | 0.730 | 0.780 |
| LambdaRank | RDKitLayer | 0.616 | 0.196 | 0.323 | 0.296 | 0.626 | 3.914 | 11.298 | 0.752 | 0.803 | 0.795 |
| LambdaRank | Desc+Morgan | 0.622 | 0.210 | 0.296 | 0.291 | 0.515 | 3.563 | 12.639 | 0.755 | 0.770 | 0.801 |

**Table S3: The ablation study of the fingerprint and descriptor effectiveness in the CTRP dataset and LRO setup.**

| Descriptor | Morgan | AtomPair | Loss  Function | AH  1_3_5 | AP  1_3_5 | NDCG  1_3_5 | AH  10_20 | AP  10_20 | NDCG  10_20 | AH  40_60 | AP  40_60 | NDCG  40_60 | CI |
| --- | --- | --- | --- | --- | --- | --- | --- | --- | --- | --- | --- | --- | --- |
|  |  | * | LambdaRank | 1.746 | 0.790 | 0.943 | 3.814 | 0.724 | 0.916 | 4.395 | 0.684 | 0.910 | 0.685 |
|  |  | * | ListAll | **1.830** | 0.788 | **0.953** | 3.954 | **0.744** | 0.919 | 4.389 | **0.716** | 0.864 | 0.682 |
| * |  |  | LambdaRank | 0.325 | 0.183 | 0.631 | 1.298 | 0.232 | 0.639 | 3.484 | 0.179 | 0.722 | **0.769** |
| * |  |  | ListAll | 0.275 | 0.155 | 0.566 | 1.088 | 0.197 | 0.594 | 3.122 | 0.156 | 0.692 | 0.759 |
| * |  | * | LambdaRank | 1.776 | **0.800** | 0.948 | 3.867 | 0.732 | **0.923** | **4.399** | 0.697 | 0.913 | 0.578 |
| * |  | * | ListAll | 1.780 | 0.768 | 0.947 | 3.922 | 0.726 | 0.915 | 4.386 | 0.696 | 0.865 | 0.553 |
| * | * |  | LambdaRank | 1.730 | 0.784 | 0.941 | 3.792 | 0.717 | 0.918 | 4.390 | 0.677 | **0.915** | **0.769** |
| * | * |  | ListAll | 1.818 | 0.779 | 0.951 | **3.957** | 0.739 | 0.919 | 4.389 | 0.711 | 0.865 | 0.683 |
|  | * |  | LambdaRank | 1.753 | 0.797 | 0.947 | 3.818 | 0.725 | 0.919 | **4.399** | 0.686 | **0.915** | 0.763 |
|  | * |  | ListAll | 1.787 | 0.772 | 0.947 | 3.939 | 0.728 | 0.917 | 4.390 | 0.701 | 0.867 | 0.685 |

**Table S4. The ablation study of the fingerprint and descriptor effectiveness in the PRISM dataset and LRO setup.**

| Descriptor | Morgan | AtomPair | Loss  Function | AH  1_3_5 | AP  1_3_5 | NDCG  1_3_5 | AH  10_20 | AP  10_20 | NDCG  10_20 | AH  40_60 | AP  40_60 | NDCG  40_60 | CI |
| --- | --- | --- | --- | --- | --- | --- | --- | --- | --- | --- | --- | --- | --- |
|  |  | * | LambdaRank | 2.812 | 0.983 | 0.993 | 8.829 | 0.924 | 0.986 | 12.581 | 0.837 | 0.968 | 0.656 |
|  |  | * | ListAll | 2.542 | 0.885 | 0.973 | 8.862 | 0.865 | 0.974 | 12.699 | 0.804 | 0.920 | 0.667 |
| * |  |  | LambdaRank | 0.544 | 0.429 | 0.855 | 1.597 | 0.384 | 0.751 | 4.833 | 0.240 | 0.702 | 0.819 |
| * |  |  | ListAll | 0.780 | 0.457 | 0.770 | 2.345 | 0.425 | 0.683 | 5.106 | 0.297 | 0.672 | 0.822 |
| * |  | * | LambdaRank | 2.799 | 0.983 | 0.993 | 8.721 | 0.920 | 0.985 | 12.540 | 0.827 | 0.966 | 0.616 |
| * |  | * | ListAll | 2.515 | 0.872 | 0.970 | 8.820 | 0.857 | 0.972 | 12.675 | 0.797 | 0.914 | 0.589 |
| * | * |  | LambdaRank | 2.804 | 0.982 | 0.993 | 8.894 | 0.924 | 0.985 | 12.588 | 0.840 | 0.967 | 0.821 |
| * | * |  | ListAll | 2.476 | 0.840 | 0.967 | 8.814 | 0.850 | 0.971 | 12.679 | 0.793 | 0.915 | 0.662 |
|  | * |  | LambdaRank | 2.795 | 0.978 | 0.991 | 8.828 | 0.920 | 0.984 | 12.584 | 0.835 | 0.966 | 0.819 |
|  | * |  | ListAll | 2.487 | 0.841 | 0.965 | 8.820 | 0.854 | 0.971 | 12.681 | 0.795 | 0.914 | 0.668 |

**Fig S23. Comparative evaluation of drug ranking behavior in the LCO setup trained on the CTRP dataset. The figure illustrates the prioritization of clinically established drugs for NSCLC and melanoma using the best and worst performing models: (a) Best model for NSCLC, (b) Best model for melanoma, (c) Worst model for NSCLC, and (d) Worst model for melanoma.**

**Fig S24. Comparative evaluation of drug ranking behavior in the LCO setup trained on the PRISM dataset. The figure illustrates the prioritization of clinically established drugs for NSCLC and melanoma using the best and worst performing models: (a) Best model for NSCLC, (b) Best model for melanoma, (c) Worst model for NSCLC, and (d) Worst model for melanoma.**

**Fig S25. Comparative evaluation of drug ranking behavior in the LRO setup trained on the PRISM dataset. The figure illustrates the prioritization of clinically established drugs for NSCLC and melanoma using the best and worst performing models: (a) Best model for NSCLC, (b) Best model for melanoma, (c) Worst model for NSCLC, and (d) Worst model for melanoma.**

**Fig S26. The plots present the sorted mean attention weights (a) and the SHAP values (b), which represent gene importance across all breast cancer cell lines in the PRISM datasets under the LCO setup. Higher values indicate a greater contribution to the model's decision-making process.**

**Fig S27. The figures illustrates the GSEA analysis of the model’s attention matrix values (a) and SHAP values (b) for cell lines in the PRISM dataset under the LCO setup, revealing key biological pathways that may influence the model's predictions.**

**Table S5. GSEA analysis of ER+ and ER- breast cancer cell lines using attention to identify key biological pathways associated with gene importance.**

| **Gene_set** | **Term** | **P-value** | **Adjusted P-value** | **Cell Line ER** |
| --- | --- | --- | --- | --- |
| MSigDB_Hallmark_2020 | Inflammatory Response | 1.21E-07 | 5.11E-06 | Positive |
| MSigDB_Hallmark_2020 | Apoptosis | 2.04E-07 | 5.11E-06 | Positive |
| MSigDB_Hallmark_2020 | mTORC1 Signaling | 1.78E-06 | 2.97E-05 | Positive |
| MSigDB_Hallmark_2020 | Cholesterol Homeostasis | 9.02E-06 | 1.13E-04 | Positive |
| MSigDB_Hallmark_2020 | TNF-alpha Signaling via NF-kB | 2.08E-05 | 2.08E-04 | Positive |
| KEGG_2021_Human | Neuroactive ligand-receptor interaction | 3.12E-26 | 5.06E-24 | Positive |
| KEGG_2021_Human | Pathways in cancer | 3.24E-26 | 5.06E-24 | Positive |
| KEGG_2021_Human | Chemical carcinogenesis | 1.07E-19 | 1.11E-17 | Positive |
| KEGG_2021_Human | Phospholipase D signaling pathway | 8.52E-19 | 6.66E-17 | Positive |
| KEGG_2021_Human | PI3K-Akt signaling pathway | 7.43E-18 | 4.65E-16 | Positive |
| MSigDB_Hallmark_2020 | Inflammatory Response | 1.15E-07 | 5.75E-06 | Negative |
| MSigDB_Hallmark_2020 | Apoptosis | 5.28E-07 | 1.32E-05 | Negative |
| MSigDB_Hallmark_2020 | mTORC1 Signaling | 1.70E-06 | 2.84E-05 | Negative |
| MSigDB_Hallmark_2020 | Cholesterol Homeostasis | 8.78E-06 | 1.10E-04 | Negative |
| MSigDB_Hallmark_2020 | TNF-alpha Signaling via NF-kB | 2.00E-05 | 2.00E-04 | Negative |
| KEGG_2021_Human | Neuroactive ligand-receptor interaction | 2.71E-26 | 4.26E-24 | Negative |
| KEGG_2021_Human | Pathways in cancer | 2.73E-26 | 4.26E-24 | Negative |
| KEGG_2021_Human | Phospholipase D signaling pathway | 7.87E-19 | 8.21E-17 | Negative |
| KEGG_2021_Human | Chemical carcinogenesis | 1.42E-18 | 1.11E-16 | Negative |
| KEGG_2021_Human | PI3K-Akt signaling pathway | 6.63E-18 | 4.15E-16 | Negative |

1.	Järvelin, K. and J. Kekäläinen, *Cumulated gain-based evaluation of IR techniques.* ACM Transactions on Information Systems (TOIS), 2002. **20**(4): p. 422-446.

2.	Manning, C.D., *An introduction to information retrieval*. 2009.

3.	Partin, A., et al., *Deep learning methods for drug response prediction in cancer: predominant and emerging trends.* Frontiers in medicine, 2023. **10**: p. 1086097.

4.	An, X., et al., *Representation of molecules for drug response prediction.* Briefings in Bioinformatics, 2022. **23**(1): p. bbab393.

5.	Gobbi, A. and D. Poppinger, *Genetic optimization of combinatorial libraries.* Biotechnology and bioengineering, 1998. **61**(1): p. 47-54.

6.	Zhong, S. and X. Guan, *Count-based morgan fingerprint: a more efficient and interpretable molecular representation in developing machine learning-based predictive regression models for water contaminants’ activities and properties.* Environmental Science & Technology, 2023. **57**(46): p. 18193-18202.

7.	Morgan, H.L., *The generation of a unique machine description for chemical structures-a technique developed at chemical abstracts service.* Journal of chemical documentation, 1965. **5**(2): p. 107-113.

8.	Rogers, D. and M. Hahn, *Extended-connectivity fingerprints.* Journal of chemical information and modeling, 2010. **50**(5): p. 742-754.

9.	Carhart, R.E., D.H. Smith, and R. Venkataraghavan, *Atom pairs as molecular features in structure-activity studies: definition and applications.* Journal of Chemical Information and Computer Sciences, 1985. **25**(2): p. 64-73.

10.	Landrum, G., *Rdkit documentation.* Release, 2013. **1**(1-79): p. 4.

11.	Mendolia, I., et al., *EMBER—Embedding multiple molecular fingerprints for virtual screening.* International Journal of Molecular Sciences, 2022. **23**(4): p. 2156.

12.	Vaswani, A., et al., *Attention is all you need.* Advances in neural information processing systems, 2017. **30**.

13.	Li, H., et al., *Network propagation predicts drug synergy in cancers.* Cancer research, 2018. **78**(18): p. 5446-5457.

14.	Cowen, L., et al., *Network propagation: a universal amplifier of genetic associations.* Nature Reviews Genetics, 2017. **18**(9): p. 551-562.

15.	Park, K., et al., *Predicting pharmacodynamic drug-drug interactions through signaling propagation interference on protein-protein interaction networks.* PloS one, 2015. **10**(10): p. e0140816.

16.	Pak, M., et al., *DGDRP: drug-specific gene selection for drug response prediction via re-ranking through propagating and learning biological network.* Frontiers in Genetics, 2024. **15**: p. 1441558.

17.	Pak, M., et al., *Improved drug response prediction by drug target data integration via network-based profiling.* Briefings in Bioinformatics, 2023. **24**(2): p. bbad034.

18.	Prasse, P., et al., *Matching anticancer compounds and tumor cell lines by neural networks with ranking loss.* NAR Genomics and Bioinformatics, 2022. **4**(1): p. lqab128.

19.	Oskooei, A., et al., *PaccMann: prediction of anticancer compound sensitivity with multi-modal attention-based neural networks. arXiv 2018.* arXiv preprint arXiv:1811.06802, 1811.

20.	Szklarczyk, D., et al., *The STRING database in 2023: protein–protein association networks and functional enrichment analyses for any sequenced genome of interest.* Nucleic acids research, 2023. **51**(D1): p. D638-D646.
